# Prevention of Intestinal Inflammation and Gut Dysbiosis by Prebiotic Grape Seed Flour in Mice with DSS-Induced Colitis

**DOI:** 10.3390/ph19081189

**Published:** 2026-07-29

**Authors:** Mohamed Mokrani, Mélanie Le Barz, Anne-Marie Elie, Élodie Renouf, Jean-Michel Mérillon, Ferid Limam, Ezzedine Aouani, André Marette, Naima Saad, Maria C. Urdaci

**Affiliations:** 1 University of Bordeaux, Centre National de la Recherche Scientifique (CNRS), Bordeaux INP, CBMN, UMR 5248, F-33600 Pessac, France; 2 Bordeaux Sciences Agro, F-33175 Gradignan, France; 3 Department of Medicine, Institut de Cardiologie et de Pneumologie du Québec, and Institute of Nutrition and Functional Foods (INAF), Université Laval, Québec, QC G1V 4G5, Canada; 4 Polyphénols Biotech-ADERA, University of Bordeaux, Bordeaux INP, Bordeaux Sciences Agro, INRAE, UMR 1366 OENO, ISVV, F-33140 Villenave d’Ornon, France; 5 Laboratory of Bioactive Substances, Center of Biotechnology of Borj Cedria, BP901, Hammam Lif 2050, Tunisia; 6 University of Limoges, LABCiS, UR 22722, F-87000 Limoges, France

**Keywords:** grape seed flour, antioxidant, anti-inflammatory, microbiota, DSS, prebiotic, *Muribaculaceae*, *Akkermansia*

## Abstract

**Background:** Inflammatory bowel disease is a complex intestinal inflammatory disorder linked to immune dysregulation, oxidative stress, and an imbalance in the gut microbiota. Grape seed flour (GSF), a winemaking by-product rich in polyphenols and fibres, displays antioxidant and anti-inflammatory properties and may help maintain gut homeostasis. **Methods:** The phenolic composition and antioxidant capacity of our GSF were evaluated. We assessed whether a diet containing 10% (*w*/*w*) GSF protects against dextran sulphate sodium (DSS)-induced acute colitis in BALB/c mice. Animals received either a standard diet or a GSF-supplemented diet before and during DSS exposure. Body weight and disease activity index were monitored. At sacrifice, colon length and colonic histology scoring were measured. The study of the faecal microbiota and predicted genome was performed using PICRUSt. Caecal metabolites, including short-chain fatty acids, were also quantified. **Results:** GSF is rich in fibres (64%) and exhibits a very high polyphenolic content and high antioxidant capacity. GSF supplementation attenuated DSS-induced weight loss and disease activity and limited colonic shortening and histological damage. It also improved biomarkers of colon, mesenteric lymph nodes (MLNs), and liver injury. At the molecular level, GSF downregulated key pro-inflammatory mediators in the colon and liver, while enhancing anti-inflammatory (e.g., IL-10) and antioxidant markers, indicating reinforcement of regulatory and redox-protective pathways along the gut–liver axis. At the gut microbiota level, GSF supplementation modulated α-diversity. Further analysis demonstrated that GSF reshaped the GM profile by preventing the blooming of some taxa, including *UBA1819*, *Akkermansia*, and *Bacteroides caecimuris*, and enriching butyrate-producing bacteria, such as *Muribaculaceae* and *Ruminococcus*. These shifts were accompanied by lower acetone and ethyl acetate levels and higher indole levels, which are known to support epithelial barrier integrity and mucosal homeostasis. **Conclusions:** Overall, GSF mitigates DSS-induced colitis through combined actions on inflammatory signalling, oxidative stress, immune regulation, microbiota composition and microbiota-derived metabolites, supporting its potential as a functional prebiotic ingredient for intestinal health.

## 1. Introduction

Inflammatory bowel disease (IBD) is a multifaceted group of medical conditions characterised by persistent inflammation of the digestive tract [1,2]. IBD affects approximately 6.8 million people worldwide and includes two main forms, Crohn’s disease (CD) and ulcerative colitis (UC), each characterised by varying degrees of inflammation and ulceration of the intestinal tract [3]. CD and UC are recognised by symptoms such as persistent diarrhoea, abdominal discomfort, rectal bleeding, weight loss, and fatigue [4], and are associated with an increased risk for colon cancer. The immune system plays a central role in the pathogenesis of IBD [5,6,7]. The gastrointestinal tract is the largest and most complex interface at which the immune system interacts with microorganisms, both beneficial and harmful [8]. The abnormal immune reaction to gut bacteria will be increased in people genetically prone to IBD. Drug management of IBD includes aminosalicylates, corticosteroids, immunosuppressants, anti-TNF blockers, the only biologic drugs approved for IBD treatment [9], and some new molecules [9]. However, treatments carry a risk of infection because they induce microbiota dysbiosis and the proliferation of pathogenic bacteria, as well as other side effects [9,10]. Because IBD management cannot be fully achieved by pharmaceutical approaches, dietary plant-based bioactives such as polyphenols, fibres, and omega-3 fatty acids have demonstrated positive effects in IBD treatment, lowering oxidative stress, increasing anti-inflammatory cytokines, decreasing pro-inflammatory cytokines, and improving dysbiosis [11,12,13].

Grape seeds exhibit a broad spectrum of pharmacological properties [14]. Grape seed flour (GSF), a source of fibres and polyphenols, possesses potential health benefits including antioxidant, anti-inflammatory, and antimicrobial properties, and may modulate the gut microbiota [15,16]. Grape seeds obtained during winemaking contain approximately 40% fibres (both insoluble and soluble), 11% protein, 16% oil, and 5–8% polyphenols (flavonoids, complex phenols, and tannins), depending on the variety [17]. Consumers use GSF as a dietary supplement for the health benefits associated with oligomeric proanthocyanidins (OPCs), the main polyphenol component. OPCs, which are otherwise known as tannin compounds, are retrieved principally in the slimy film that surrounds the seeds [14,18]. OPCs are the oligomers and polymers of flavan-3-ol (with degrees of polymerisation ranging from 2 to >15). In grape seeds, the identified OPCs are catechins, epicatechins, and their gallic acid esters, which serve as the principal monomers, together with various dimeric, trimeric, and tetrameric procyanidins [19]. Important, the polyphenolic composition of grape seeds is highly dependent on the grapevine variety, environmental factors, and the winemaking process [20]. For a few years, dietary polyphenols have been considered as prebiotics for their ability to reshape the gut microbiota (GM) balance [21,22]. Importantly, it must also be considered that the GM can metabolise grape seeds’ polyphenols into other bioactive, low-molecular-weight compounds [23]. GSF polyphenol content, along with the large quantity of fibres, can contribute greatly to their prebiotic effects. GSF can help mitigate inflammation and oxidative stress, which are associated with various chronic diseases, including IBD and irritable bowel syndrome (IBS). Pistol et al. [24,25] demonstrated in a model of intestinal inflammation using DSS-treated piglets that a diet enriched with 8% GSF can restore mucosal barrier function and pro-inflammatory and innate immune markers. The addition of GSF to healthy diets also induced health benefits—for instance, by extending longevity in rats. These effects were mainly due to its antioxidant and anti-inflammatory properties [26,27].

The DSS-induced murine colitis model is one of the most widely used and well-validated preclinical tools for studying the physiopathology of IBD. Its mechanism of action involves direct disruption of the intestinal epithelial barrier, leading to increased mucosal permeability, activation of the innate immune system, and a cascade of pro-inflammatory signals that closely recapitulate hallmarks of UC in patients [28]. It is therefore particularly well-suited for investigating the preventive potential of bioactive dietary compounds, such as GSF, administered prior to colitis induction.

Furthermore, most research on the anti-inflammatory effects of grape seed polyphenols has been conducted using polyphenolic extracts. For example, the consumption of grape pomace extract or grape seed proanthocyanidin extract in a murine DSS-induced colitis model improved colon inflammation and the composition of the microbial community [29,30,31]. GSF polyphenols could have synergistic effects with other grape seed phytochemicals, such as fibres and polyunsaturated fatty acids (PUFAs). In the present study, we aimed to investigate the impact of a GSF in a DSS model of colitis, focusing on the effects on the GM, inflammation, and the mucus barrier.

## 2. Results

### 2.1. Grape Seed Phenolic Compound Characterisation

#### 2.1.1. Characterisation and Quantification of Phenolic Compounds (Excluding Anthocyanins)

Phenolic compounds in GSF PP extract were identified and quantified using UHPLC-DAD-MS. All results are summarised in Table 1. Flavan-3-ols represent the dominant class of phenolic compounds of GSF extract. Catechin, epicatechin, and epicatechin-3-O-gallate exhibited the highest concentrations among the identified molecules (4.16 mg/g), confirming that monomeric and galloylated flavan-3-ols are the major contributors to the GSF phenolic pool. These results are consistent with previous reports on grape seed polyphenols [32,33]. Type B procyanidin dimers and trimers, and their galloylated derivatives, are also detected in substantial amounts (2.01 mg/g), indicating the presence of oligomeric procyanidins that complement the monomeric epicatechin units and further enrich the flavonoid fraction of the extracts. In addition to flavan-3-ols, several low-molecular-weight phenolic acids were identified, including gallic acid, protocatechuic acid, and coumaric acid. These acids are present in smaller quantities than flavonoids but contribute to the overall antioxidant capacity and chemical diversity of GSF.

#### 2.1.2. Characterisation and Quantification of Anthocyanins

The anthocyanins in the GSF extract were characterised and quantified by UPLC-DAD-MS. The results, summarised in Table 2, show that the anthocyanin content (1.3 mg/g of extract) accounts for roughly 13% of the total polyphenolic compounds (TPPs) quantified in the GSF extract (10.25 mg of TPP/g of extract).

#### 2.1.3. Antioxidant Activity

The ORAC assay determined the antioxidant capacity of GSF. The extract showed a value of 1.68 ± 0.003 g gallic acid equivalents per 100 g of GSF extract, indicating its high free-radical-scavenging potential. This strong activity is consistent with the high levels of flavan-3-ols and other phenolic compounds quantified in the GSF extract. Similar ORAC values have been reported for grape seed flavanol fractions obtained by comparable extraction procedures, confirming the robustness of our findings [32,33].

#### 2.1.4. Lipophilic Compounds and Fibre Content Determination

The chemical analysis of GSF is illustrated in Table 3. The GSF contains 9.5% fat and 64% fibre.

### 2.2. GSF Alleviates DSS-Induced Colitis Symptoms in Mice

**Figure 1:**
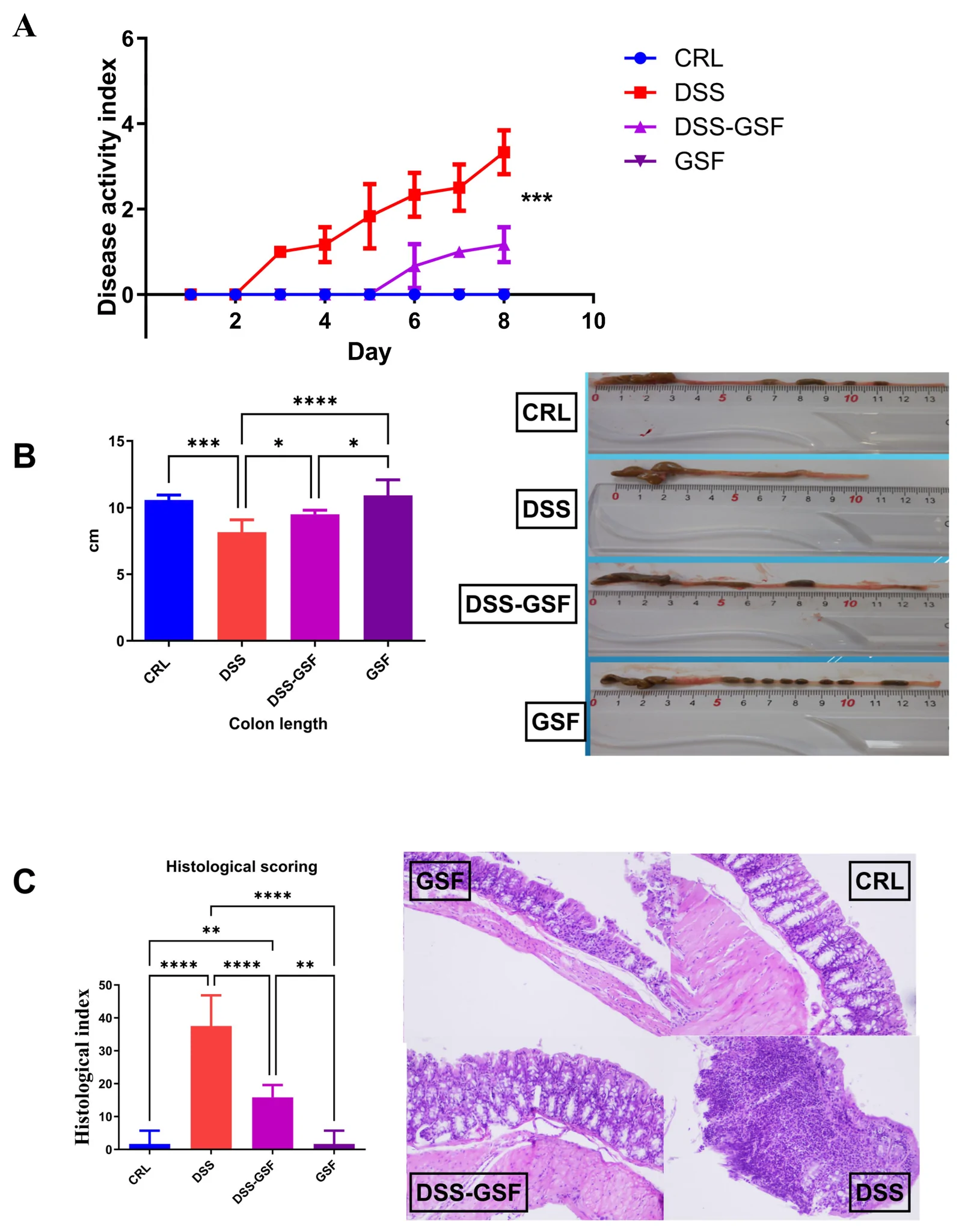
Amelioration of DSS-induced colitis by dietary supplementation with GSF. (**A**) The disease activity index during the DSS administration was calculated with weight loss, stool consistency, and rectal bleeding. (**B**) Colon length was measured in colitis-induced mice on day 19. (**C**) Sections of the colon were stained with haematoxylin and eosin. The histological score was calculated from the severity of inflammation, the thickness of inflammatory involvement, epithelial damage, and the extent of the lesion. Images were acquired at 40× magnification; scale bar = 100 µm. Data are the mean ± SEM from three independent experiments (n = 6). Statistical significance was set at *p* < 0.05 (* *p* < 0.05, ** *p* < 0.01, *** *p* < 0.001, **** *p* < 0.0001).

Regarding physiological parameters, there were no significant differences in food and water consumption between cages or intervention groups. The colitis was successfully induced through the administration of DSS for one week. To assess the ameliorative effect of GSF, we monitored the DAI scores and colon length in mice across groups (Figure 1A,B). DAI encompasses factors such as body weight loss, faecal consistency, and haematochezia [35]. Like an elevated DAI score, colon shortening is a good indicator of inflammation in DSS-induced colitis models [28]. Here, DSS significantly increased DAI and decreased colon length (Figure 1A–C) as compared with the CTL group without DSS. However, GSF significantly improved the DAI score (Figure 1A) and partly prevented the reduction in the colon length normally induced by DSS (Figure 1B). In parallel, the GSF group without DSS was still similar to the control group, indicating that GSF alone does not have any negative effect in this context.

### 2.3. GSF Substantially Alleviated the Colonic Tissue Damage

H&E staining of colonic sections from DSS-treated mice showed severe inflammatory injuries, with marked crypt distortion, pronounced submucosal oedema, and dense immune cell infiltration (Figure 1C).

In contrast, GSF supplementation markedly reduced these DSS-induced lesions and largely preserved colonic morphology. Most of the epithelial lining remained continuous, and the mucosal architecture was predominantly intact, indicating substantial histological protection by GSF.

#### 2.3.1. Colon Tissue

**Figure 2:**
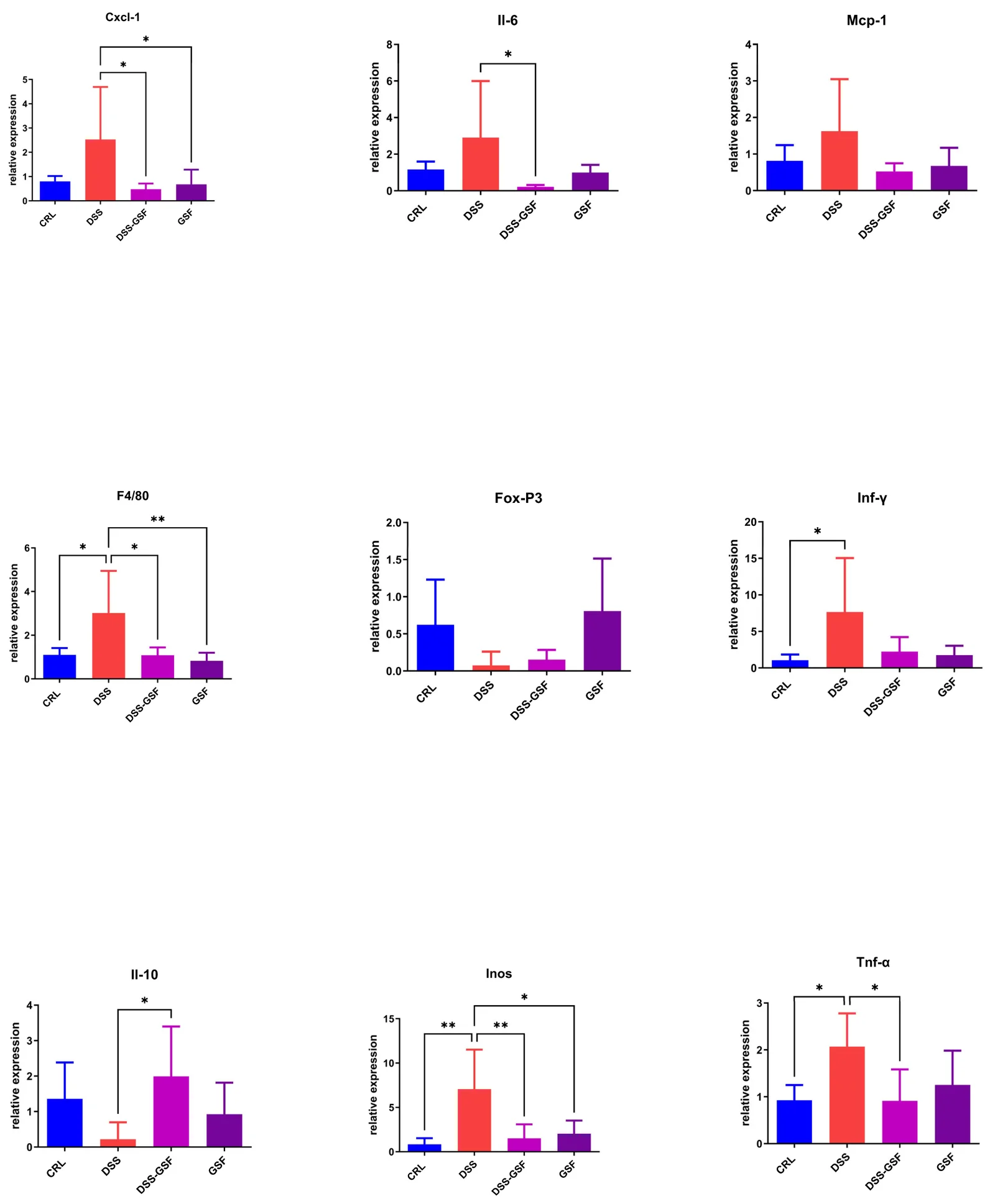
Effect of the GSF-supplemented diet on gene expression in the colon of colitis-induced mice. Transcripts of Cxcl1, Il-6, Mcp1, F4/80, Foxp3, Ifn-γ, Il-10, iNOS, Tnf-α, and in the colon of colitis-induced mice fed with the control or GSF diet were measured by quantitative PCR: CRL: control group, in blue; GSF: GSF-only group, in mauve; DSS: DSS-only group, in red; DSS-GSF in pink. Data are expressed as mean ± SEM (n = 6). Statistical significance was set at *p* < 0.05 (* *p* < 0.05, ** *p* < 0.01).

DSS treatment markedly increased colonic CXCL1 chemokine, indicating enhanced leukocyte recruitment to the inflamed mucosa. It also upregulated IL-6, indicating activation of a pro-inflammatory cytokine network. IFNγ was similarly induced, indicating a strengthened Th1-type effector response in the colon. INOS expression increased in parallel with nitrosative stress and epithelial injury. However, GSF supplementation counteracted this inflammatory program in DSS-treated mice. It significantly reduced CXCL1, IL-6, IFNγ, F4/80, and INOS expression, suggesting attenuated immune-cell recruitment and effector activation (Figure 2). In contrast, IL-10 expression, an anti-inflammatory cytokine, was significantly increased in the DSS-GSF group compared with the DSS-alone group. This shift toward higher IL-10 levels is consistent with strengthened regulatory pathways that support the resolution of inflammation and the restoration of mucosal homeostasis (Figure 2).

#### 2.3.2. Liver

**Figure 3:**
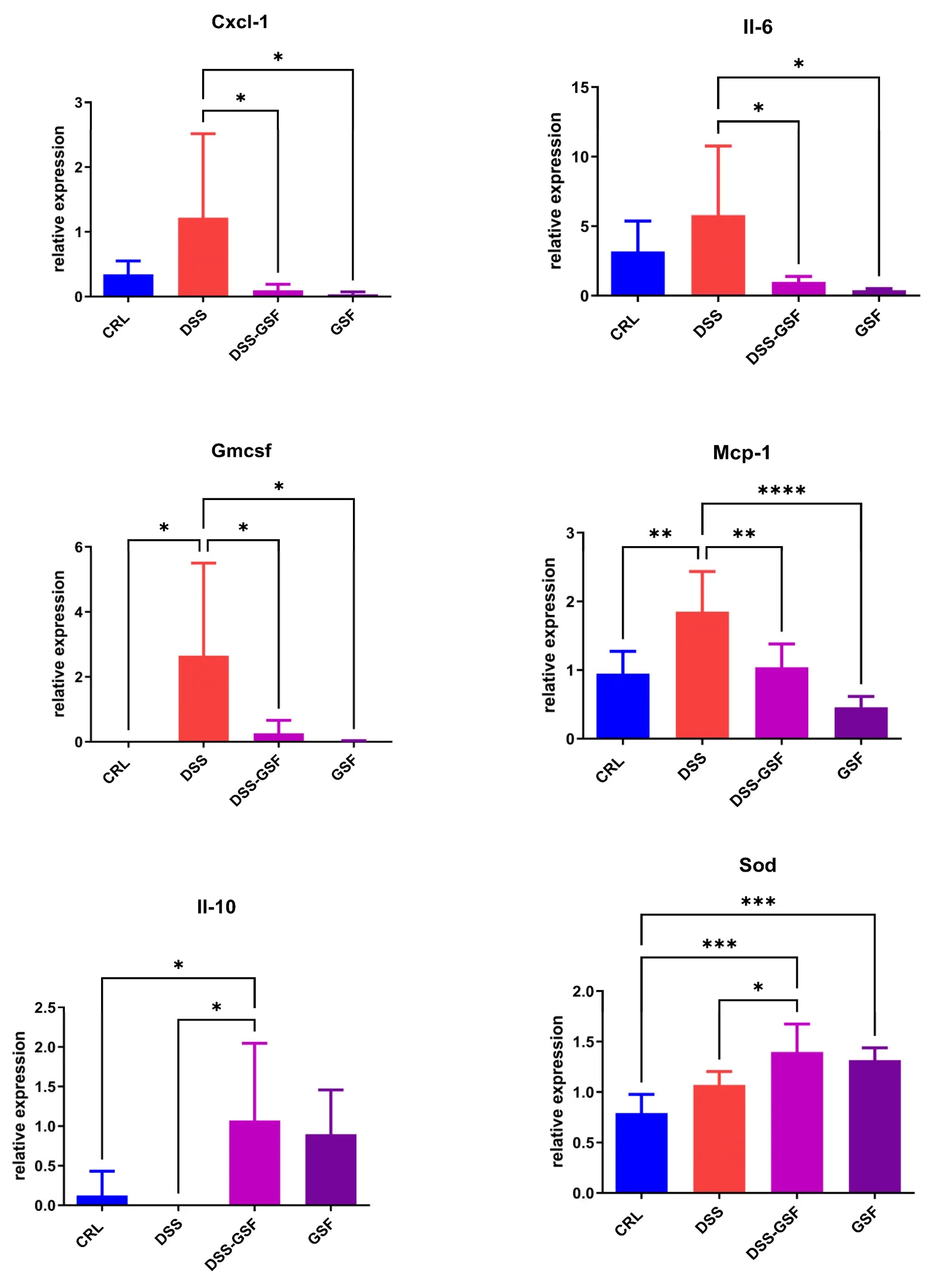
Effect of the GSF-supplemented diet on gene expression in the liver of colitis-induced mice. Transcripts of Cxcl1, Il-6, Gmcsf, Mcp1, Sod1, and Il-10 in the colons of colitis-induced mice fed the control or tannin. CRL: control group, in blue; GSF: GSF-only group, in mauve; DSS: DSS-only group, in red; DSS-GSF in pink. Data are expressed as mean ± SEM (n = 6). Statistical significance was set at *p* < 0.05 (* *p* < 0.05, ** *p* < 0.01, *** *p* < 0.001, **** *p* < 0.0001).

DSS treatment significantly increased hepatic CXCL1, MCP1 and GM-CSF expression, genes involved in the recruitment and activation of inflammatory monocytes and macrophages in the liver (Figure 3). In contrast, GSF supplementation reduced the expression of CXCL1, MCP1, GM-CSF, and IL-6 in DSS-treated mice (Figure 3), indicating weaker chemokine signalling and reduced neutrophil and monocyte influx into hepatic tissue. GSF also increased IL-10 expression, a gene coding for a cytokine known to limit T-cell- and macrophage-driven liver injury and fibrosis. Finally, SOD expression was elevated in the DSS-GSF group, consistent with enhanced antioxidant enzyme activity and better control of reactive oxygen species in the liver.

#### 2.3.3. Mesenteric Lymph Nodes (MLNs)

**Figure 4:**
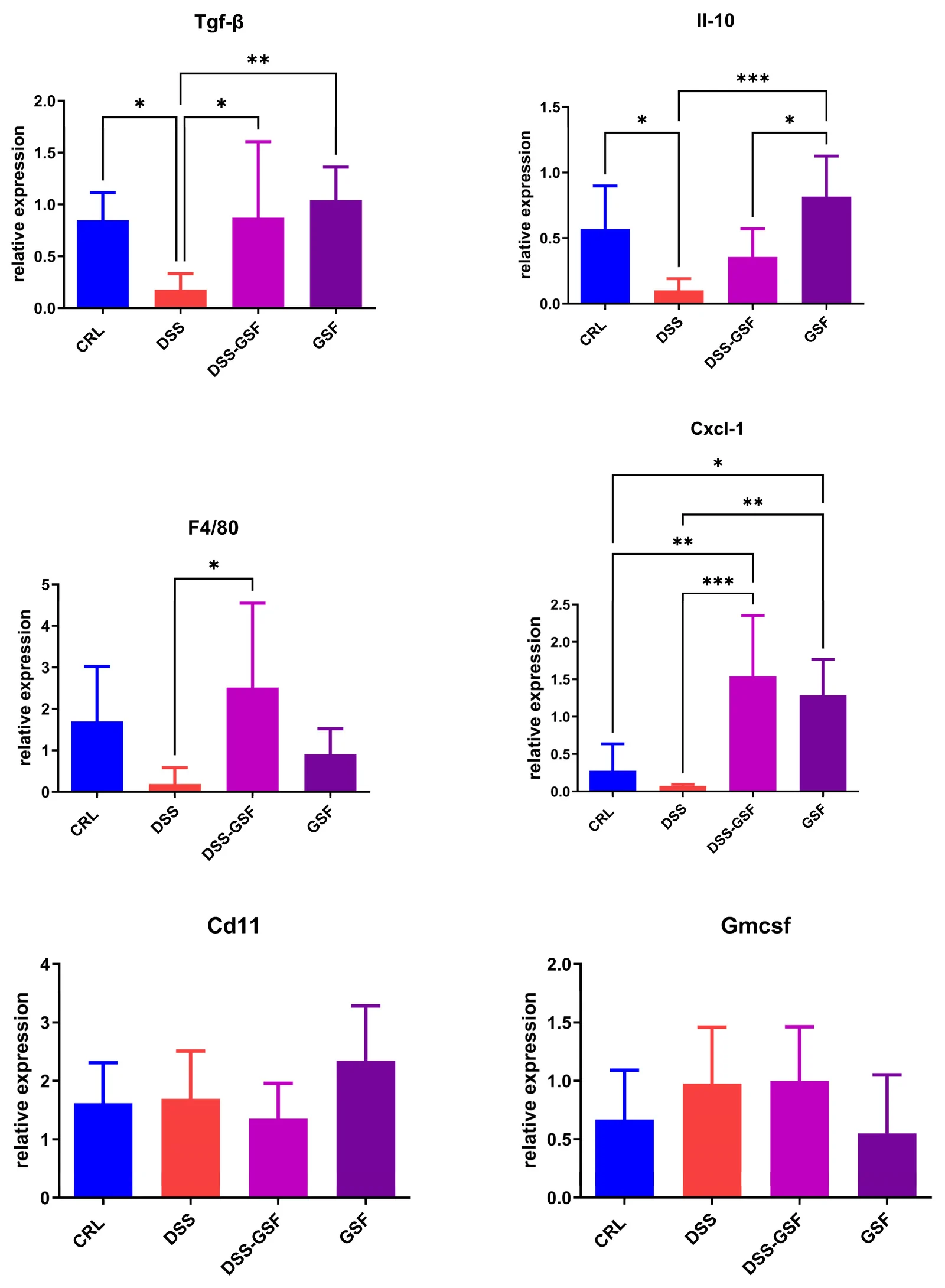
Effect of the GSF-supplemented diet on gene expression in MNL of DSS-induced colitis in mice. Transcripts of Tgf-β, Il-10, F4/80, Cxcl1, Cd11 and Gmcsf in the MNL were measured by qPCR. CRL: control group, in blue; DSS: DSS-only group, in red; DSS-GSF in pink; and GSF: GSF-only group, in mauve. Data are expressed as mean ± SEM (n = 6). Statistical significance was set at *p* < 0.05 (* *p* < 0.05, ** *p* < 0.01, *** *p* < 0.001).

DSS treatment significantly reduced TGFβ and IL-10 expression (Figure 4), indicating an altered regulation of cytokine signalling. This pattern is consistent with an impaired immunoregulatory milieu and weakened tolerogenic responses under DSS challenge. Herein, GSF supplementation positively prevents these negative effects, significantly increasing TGFβ, IL-10, and F4/80 expression compared with the DSS group (Figure 4). This suggests a regulation of cytokine networks and macrophage-associated functions, supporting immune homeostasis in the MLNs.

### 2.4. Microbiota Analysis

**Figure 5:**
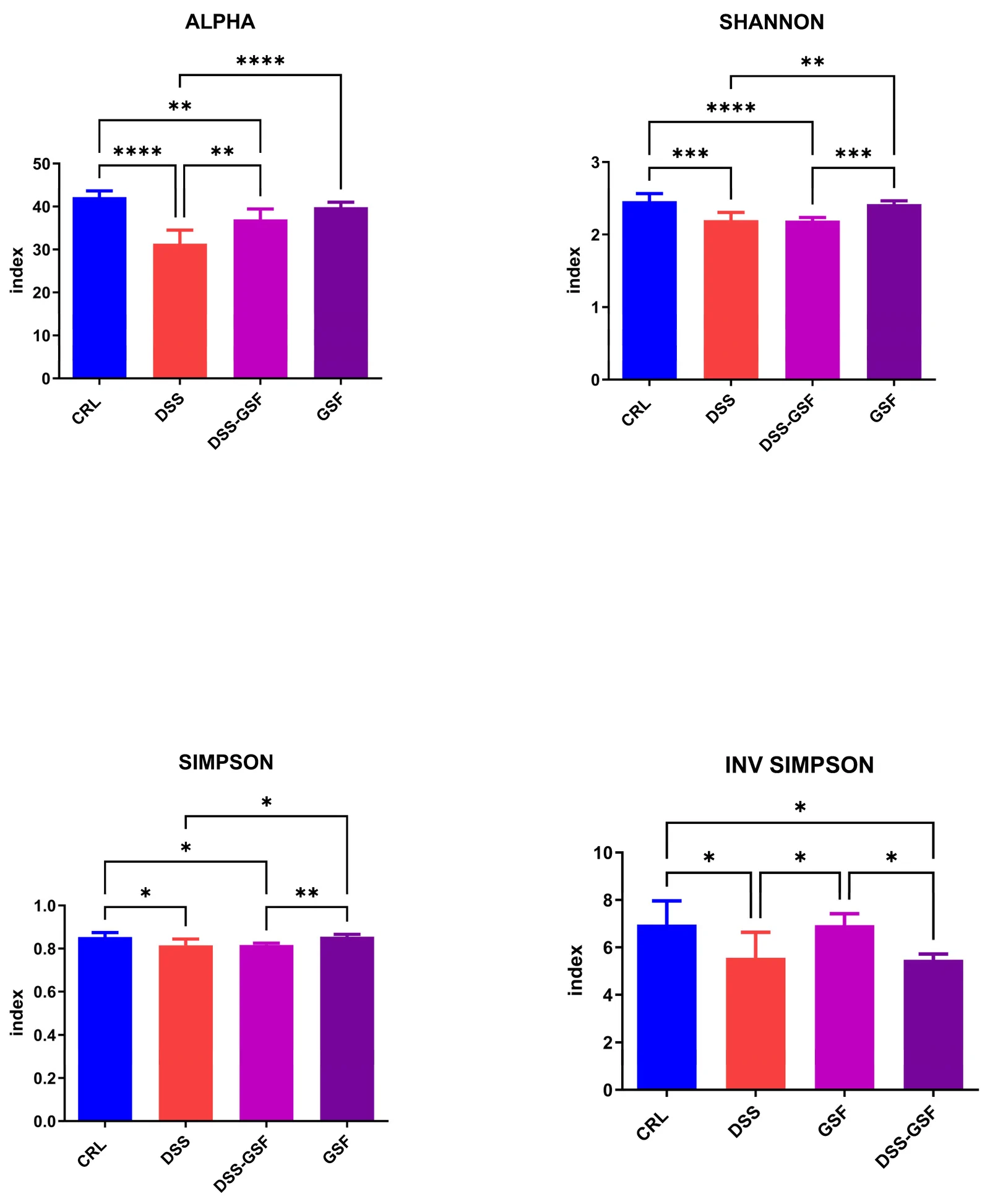
Effects of GSF, DSS and DSS administered with GSF on microbiota alpha diversity, including Shannon diversity, Simpson diversity and inverse Simpson diversity. CRL: control group, in blue; GSF: GSF-only group, in mauve; DSS: DSS-only group, in red; DSS-GSF in pink. Data are expressed as mean ± SEM (n = 6). Statistical significance was set at *p* < 0.05 (* *p* < 0.05, ** *p* < 0.01, *** *p* < 0.001, **** *p* < 0.0001).

**Figure 6:**
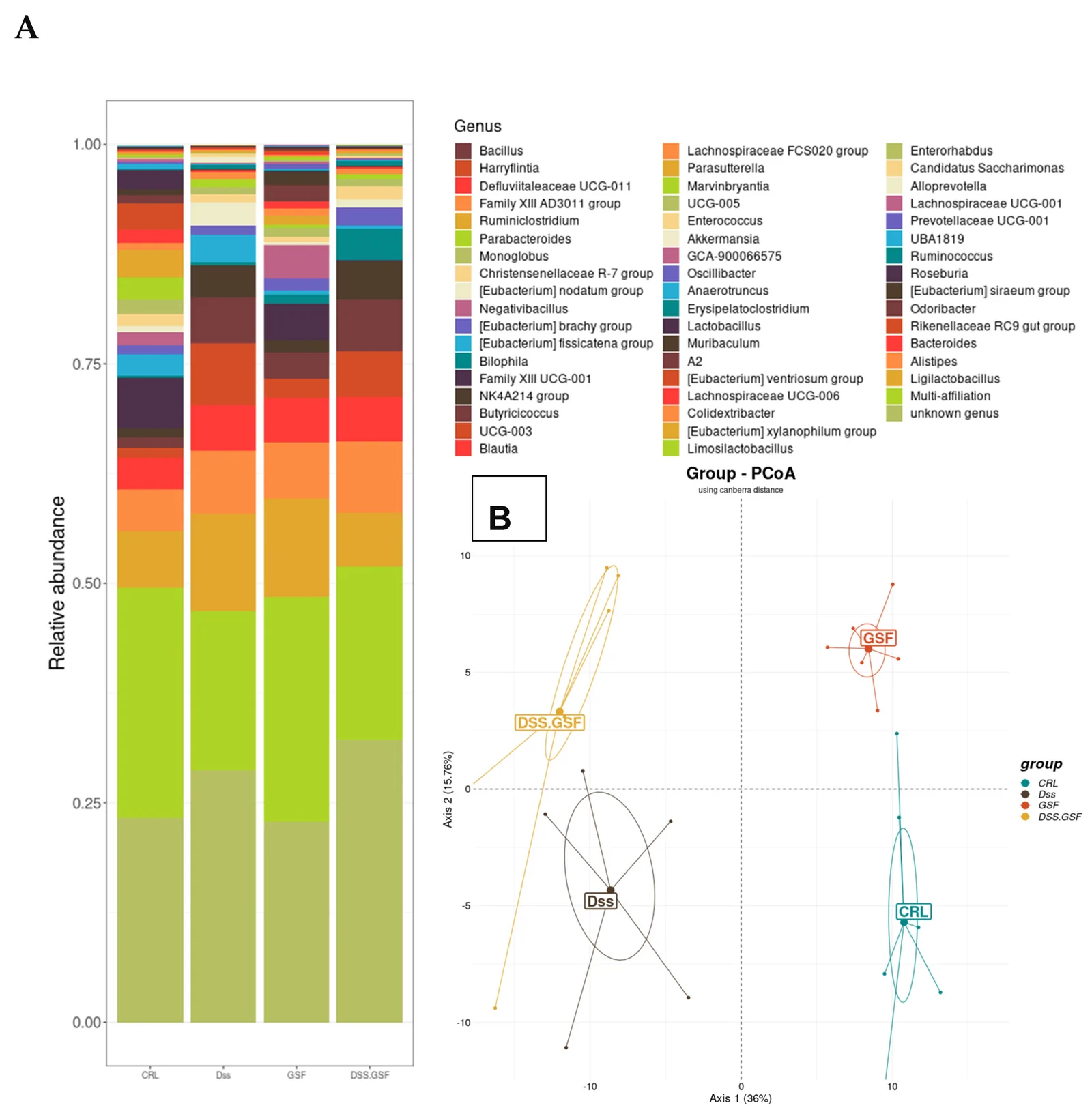
Taxonomic composition of the gut microbiota under different treatments at the genus level (n = 6). (**A**) Bar plot of the proportions of various taxa in the different groups (CRL, GSF, DSS, DSS + GSF). (**B**) Principal coordinate analysis using Canberra distance; Axis 1:15.76%, Axis 2: 36%; *p*-value: 0.001.

The Illumina MiSeq platform was used to analyse changes in the gut microbiota associated with DSS-induced colitis and the effects of the GSF treatments on the gut microbiota (GM). A total of 485,718 sequencing reads for the faecal microbiota were analysed using GALAXY [36] and SHAMAN [37]. Alpha diversity, which denotes the overall microbial diversity within each sample, was assessed at the genus level. To characterise this diversity consistently, Shannon–Simpson and inverse Simpson indices were computed. At the genus level, the DSS treatment significantly decreased alpha diversity compared to the control group; in contrast, the alpha diversity in the DSS-GSF-treated mice was significantly enhanced compared to the DSS group (Figure 5). The Shannon, Simpson, and inverse Simpson indices were significantly reduced by the DSS process. Still, the GSF did not improve them (Figure 5). Moreover, as represented in the PCoA Canberra distance plots (Figure 6B), at the genus level, the GM populations showed a significant separation among the four mice groups (*p* < 0.001), indicating changes in the GM after the three treatments. Specific modifications characterised these microbiota profiles. We have taken into consideration the modifications concerning bacteria with an abundance of 0.5% or greater.

**Figure 7:**
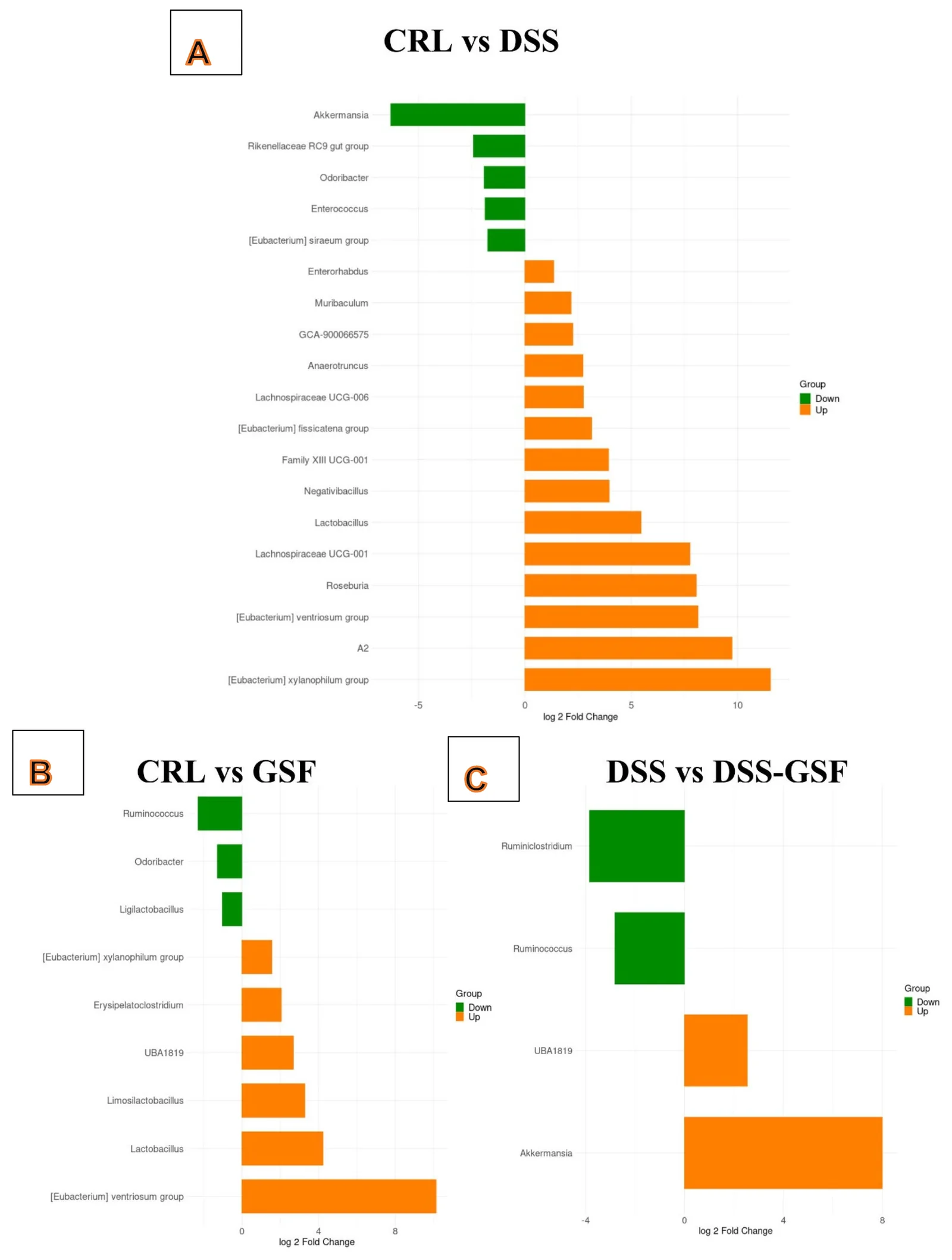
Log_2_ fold changes in microbial taxa abundance across experimental contrasts at the genus level (n = 6). Panels depict pairwise comparisons: (**A**) (CRL) vs. DSS, (**B**) (CRL) vs. (GSF), and (**C**) DSS vs. DSS-GSF. Bar colours indicate the direction and significance of change: green denotes genera significantly enriched, and orange denotes genera significantly depleted (*p* < 0.05).

At the phylum level, the different interventions did not modulate the more abundant phyla. However, DSS treatment significantly increased Desulfobacterota and Verrucomicrobiota and decreased Actinobacteriota (Figure S1). At the family level (from most to least represented families), DSS increased *Rikinellaceae*, *Marinifilaceae*, *Desulfovibrionaceae*, *Akkermansiaceae*, *Erysipelatoclostridiaceae*, and *Enterococcaceae*, and decreased *Eggerthellaceae* (Figure S2). In addition, at the genus level, DSS significantly increased the relative abundance of seven genera that are considered harmful in certain circumstances, such as *Rikinellaceae* RC9, *Odoribacter*, *Eubacterium siraeum* group, *Akkermansia*, and *Enterococcus*. In parallel, the relative abundance of several genera decreased, including beneficial bacteria such as *Lactobacillus*, *Muribaculum*, and various SFCA producers (*Roseburia*, A2, *Eubacterium*, *Lachnospiraceae* UGC 001, and UGC 006), indicating severe dysbiosis (Figure 7A). Interestingly, the addition of GSF to the diet ameliorated dysbiosis by maintaining the relative abundance of some beneficial bacteria such as *Muribaculaceae* and *Ruminococcus* and lowering the representation of potentially harmful bacteria such as *UBA1819*, *Akkermansia,* and *Bacteroides caecimuris* in the DSS-GSFgroup (Figure 7C and Figure S2).

In addition, by comparing the control and GSF groups, we found that GSF could modify various bacterial groups (Figure 7B and Figure S2). *Ligilactobacillus* increased considerably in the GSF group concurrently with a decrease in *Lactobacillus* and *Limosilactobacillus.* Moreover, *Odoribacter* (from the *Marinifilaceae* family) and *Ruminococcus* increased in the GSF group, whereas potential pathogens, such as *UBA1819*, *Erysipelatoclostridium*, and *B. caecimuris*, decreased (Figure 7B and Figure S2).

Interestingly, colon inflammatory biomarkers, colon length, and VOCs in DSS-induced colitis mice treated with the GSF diet showed significant positive or negative correlations with the relative abundance of different microbiota groups, as determined by Spearman’s correlation analysis. In particular, *Muribaculaceae* presented opposite correlations compared to *Akkermansia* and *B. caecimuris*. The acetone content in faeces was positively correlated with IL-6 (r = 0.76), Akkermansia (r = 0.72), and B. caecimuris (r = 0.66), and negatively correlated with colon length (r = −0.76) and *Muribaculaceae* (r = −0.42) (Table S3). The presence of indole in faeces was only positively correlated with Ruminococcus. *UBA1819* showed no significant correlations with the measured biomarkers. Complete correlation data are provided in Table S3. Similar correlation results are observed for the parameters measured in the liver. In MLN, *Muribaculaceae* correlated positively with IL-10 and TGF-β (r = 0.53, 0.56), *Akkermansia* correlated negatively with IL-10 and TGF-β (r = −0.55, −0.64), and *B. caecimuris* correlated negatively with TGF-β (r = −0.57) (Table S3).

### 2.5. Gene Function Analysis of the Gut Microbiota

**Figure 8:**
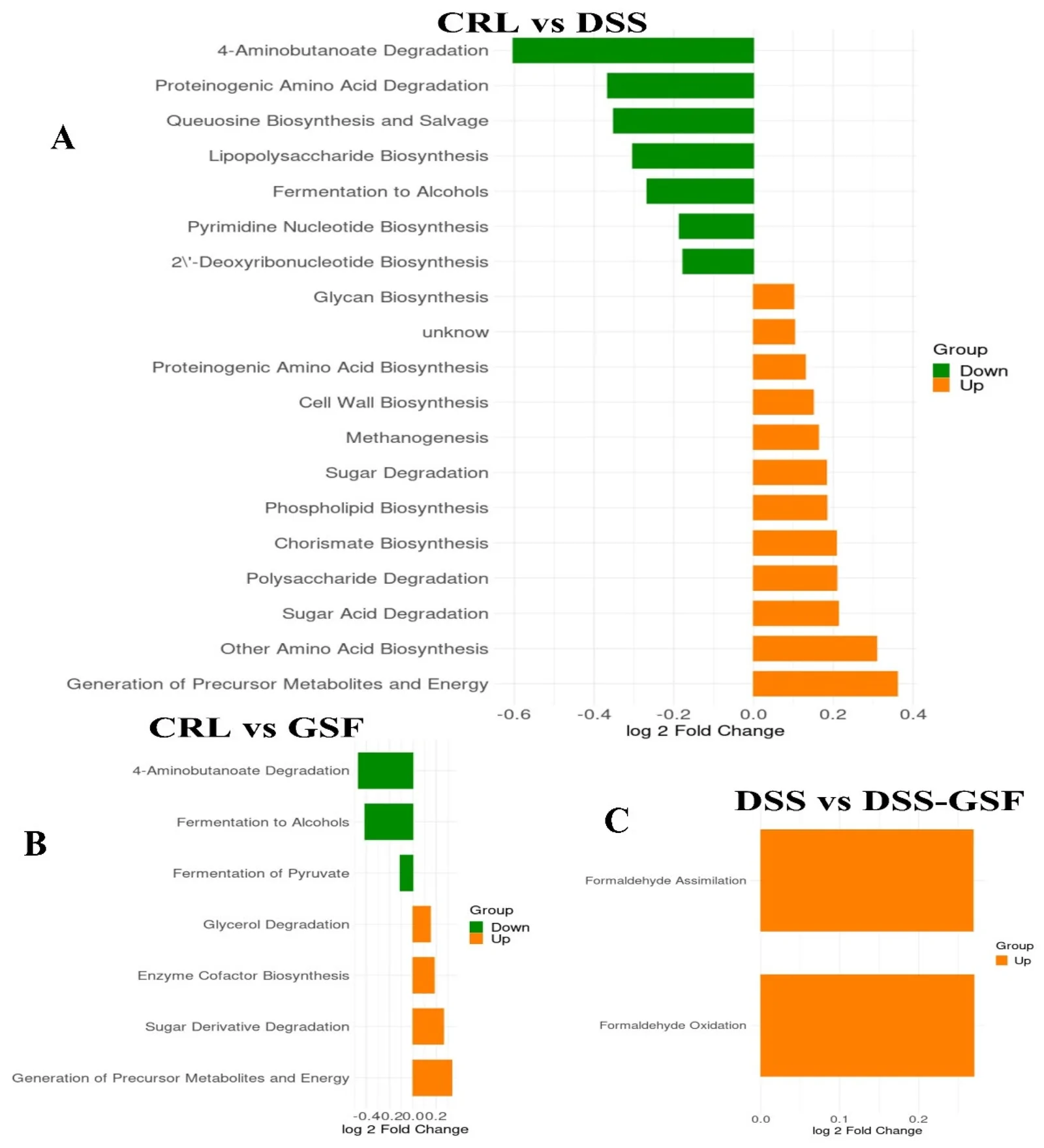
Log2 fold changes in predicted microbiota abundance PICRUSt using KEGG for different experimental contrasts (n = 6). Panels depict pairwise comparisons: (**A**) (CRL) vs. DSS, (**B**) (CRL) vs. (GSF), and (**C**) DSS vs. DSS-GSF. Green—significantly increased, and orange—significantly decreased (*p* < 0.05).

Using PICRUSt2, we predicted the functional potential of bacterial communities and analysed functional differences among the four groups. These predictions are supported by integration into (MtaCyc), (KO), (PFAM), (COG), (TIGRFAM), (PHENO) and (EC) numbers to explore related functional changes. As shown in Figure 8A, a marked number of predicted microbial metabolic functions were modified after DSS treatment, reflecting the GM profile modifications already reported. Pathways linked to inflammatory activation were relatively enriched in DSS-treated samples. In contrast, pathways typically associated with anabolic and barrier-supportive functions, such as proteinogenic amino acid biosynthesis, queuosine biosynthesis and salvage, glycan and polysaccharide degradation, alcohol fermentation, and 2′-deoxyribonucleotide biosynthesis, were relatively depleted, indicating a loss of saccharolytic and metabolically versatile taxa under DSS-induced inflammatory stress. However, most of the observed changes between the DSS and DSS-GSF groups were limited, with increases in phosphorus compound metabolism and decreases in formaldehyde oxidation and formaldehyde assimilation in the DSS + GSF group (Figure 8C). Formaldehyde is a toxic agent with a high inflammatory potential. Moreover, the GSF-enriched diet favoured increased alcohol and pyruvate fermentation compared to the control.

### 2.6. Analysis of Volatile Organic Compounds (VOCs)

**Figure 9:**
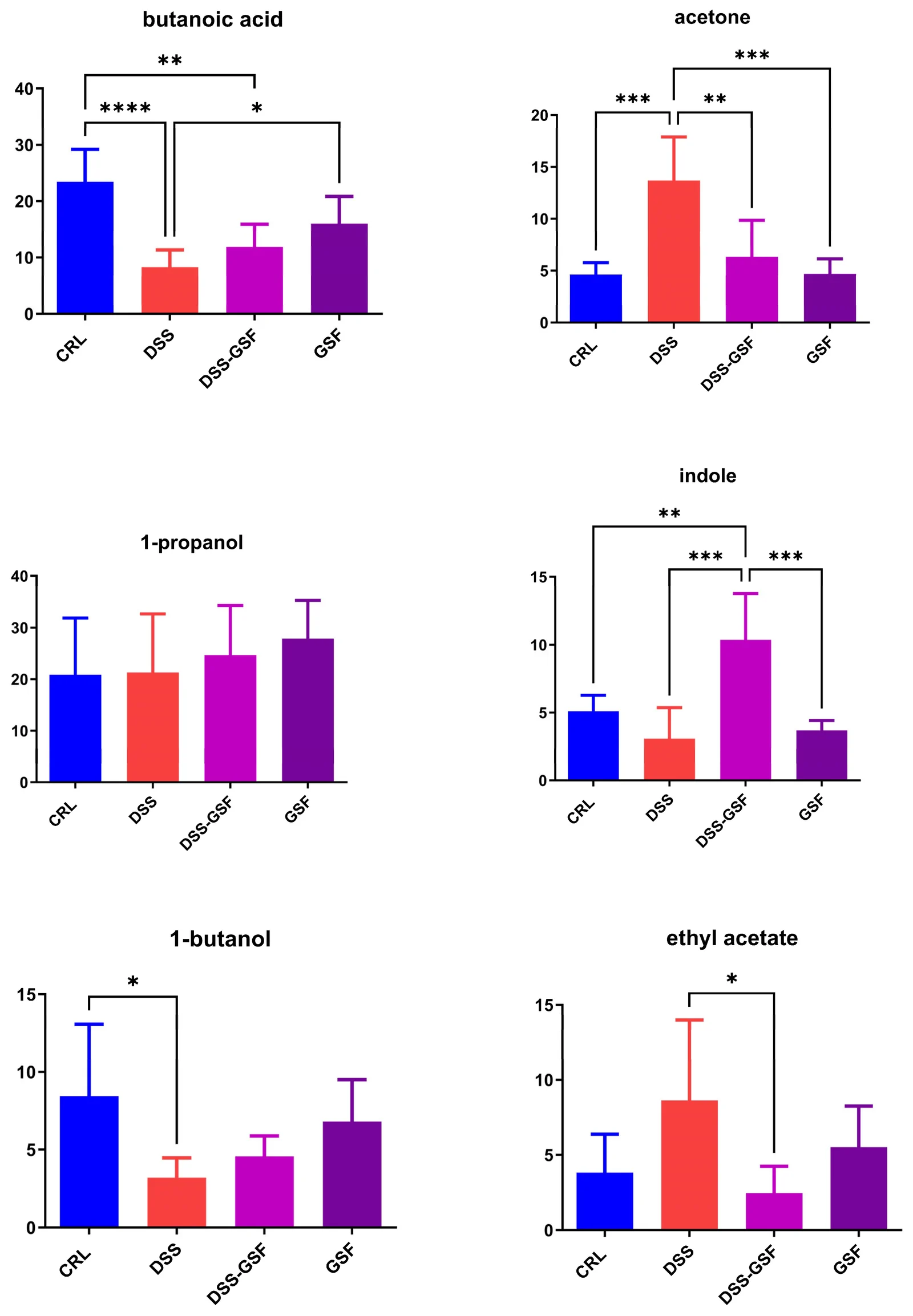
GSF supplementation affected faecal short-chain fatty acid (SCFA) generation in DSS-treated mice. CRL: control group, in blue; GSF: GSF-alone group, in mauve; DSS: DSS-alone group, in red; DSS-GSF administration group, in pink. Data are expressed as mean ± SEM (n = 6). Statistical significance was set at *p* < 0.05 (* *p* < 0.05, ** *p* < 0.01, *** *p* < 0.001, **** *p* < 0.0001).

In DSS-treated mice, the faecal metabolite profile shifted towards an unfavourable profile (Figure 9). Compared with controls, DSS markedly reduced the levels of butanoic acid (butyrate) and 1-butanol, a metabolite that can be derived from butyrate. Concomitantly, DSS increased the levels of acetone and ethyl acetate, metabolites often associated with dysbiosis, inflammation, and metabolic stress.

This imbalance was only partially prevented by the GSF supplementation. Indeed, in DSS animals receiving GSF, butanoic acid and 1-butanol tended to recover toward control values, and, interestingly, the acetone and ethyl acetate levels were significantly reduced. Moreover, the indole concentration was significantly elevated in the DSS-GSF group compared to the other groups.

## 3. Discussion

In this work, we examined whether supplementing the diet with complex bioactive compounds from grape seed could help alleviate the typical inflammatory response associated with IBD in a well-established DSS-induced colitis model. We obtained the GSF from the red grape cultivar Carignan, cultivated in Tunisia. GSF demonstrated high antioxidant potential, as evidenced by its high ORAC value, and possesses a phenolic composition dominated by catechins (23.2%), epicatechin (12%), and gallic acid (18%), and B-type procyanidins, dimers and trimers (21%). The anthocyanins represented 11.90%, and are predominantly Malvidin-3-O-(6-O-p-coumaroyl)-glucoside. This phenolic composition, important for health benefits, is generally within the ranges reported for other varieties but remains unique [17,38,39]. Fibres are high in the GSF (64%), in accordance with previous studies, and the majority are insoluble (IF), mostly composed of cellulose and hemicelluloses, since soluble fibres in the seeds are present at very low concentrations [39]. In a DSS mouse model, cellulose improved colitis symptoms, stimulated IL-10 production, while decreasing Tnf-α, and restored the gut microbiota [40]. Hemicellulose alleviated an impaired gut barrier, microbiome dysbiosis, and reduced colonic inflammation in obese mice [41]. These IF could contribute to the positive effect of the GSF in our study. We can also consider the presence of non-extractable polyphenols (NEPs) in grape seeds, which are carried via fibres and can confer a positive effect [39,42]. The GSF also contained 9.5% fat, which is within the literature range [17]. Grape seed oil contained up to 75.5% PUFAs—in particular, linoleic acid, vitamin E, and phytosterols—which may have a synergistic role with the other phytochemicals already mentioned [17].

In a previous study, we demonstrated the positive effect of a grape seed extract on inflammation [30]. In the present study, we investigated the effect of adding 10% grape seed flour to the diet. Each mouse receives a provision from GSF: 2.6 mg of polyphenols, 28.5 mg of fat, and 195 mg of fibres. Similar doses of GSF in the diet have demonstrated positive effects across different animal models [24,26]. In our study, GSF diet supplementation had a significant anti-inflammatory effect in mice. Similar to Pistol et al. (2020), ref. [24], who used a piglet’s DSS model, we demonstrated a significant improvement in physiological and histological parameters in our model. Compared with our last study, GSF and GSE supplementation had a similar effect on reducing inflammatory symptoms [30]. Some plant-derived polyphenols show clear anti-inflammatory activity by interfering with key signalling pathways, such as NF-κB, and by reshaping cytokine and chemokine secretion patterns such as IL-6, IFN-γ, CXCL1, IL-10, as is the case with our GSF, rich in polyphenols but also in fibres [43,44,45,46].In DSS-induced colitis, primary intestinal inflammation leads to secondary metabolic alterations in the liver. We observed an increase in the inflammatory markers CXCL1, MCP1, GM-CSF and IL-6 in the liver with DSS and a decrease with GSF treatment, supporting the concept that intestinal injury can affect liver function along the gut–liver axis [43], and that GSF potentially protects against extra-intestinal manifestations of IBD [44]. Also, GSF treatment increased the expression of the antioxidant gene SOD1. The reduction in inflammation and oxidative stress in the gut–liver axis is the keystone of IBD treatment [45]. In mesenteric lymph nodes, DSS alone markedly reduces the expression of the regulatory cytokines TGF-β and IL-10 compared with controls, indicating a loss of immunoregulatory tone during colitis, whereas GSF treatment restores and even enhances their expression and is accompanied by increased F4/80. The results suggest an accumulation of macrophages with a more regulatory phenotype, while GM-CSF remains largely unchanged, indicating that GM-CSF selectively reinforces anti-inflammatory rather than general myelopoietic pathways [46].

The GM plays a central role in the inflammation and oxidation implicated in IBDs [1]. The GM and its metabolites can regulate immune cells and maintain mucosal immunity, including immune cell maturation, differentiation, and function [47]. In our study, the DSS treatment dramatically altered the GM composition and functions. GM diversity indices were decreased by the DSS treatment. With GSF, which increases the diet’s fibre content, a marked improvement in diversity parameters could be expected, but only a slight impact was observed. However, the use of GSE treatment with the highest concentration of polyphenols was more efficient in terms of restoring diversity parameters [30].

Despite the slight impact on diversity, PCoA revealed distinct clustering of GM profiles among the different intervention groups. Major GM modifications were induced by DSS, with the increase in harmful bacterial groups and the important decrease in more beneficial bacteria—characteristics of IBDs [1]. Compared with DSS alone, GSF supplementation only had a minor effect on these bacterial groups, which is contrary to what we previously reported in our last study using a purified extract of grape seeds OPCs [30].

However, various interesting modulations have been retrieved. GSF supplementation significantly increased the abundance of the *Muribaculaceae* family and the *Ruminococcus* genus. The alleviating effects of a plant-based diet on IBD, obesity, and type 2 diabetes are associated with an increased abundance of *Muribaculaceae*, a group of bacteria that is negatively associated with gut inflammation [48,49], as was also found in DSS-induced inflammation in animals [30,50,51]. It is interesting to note that they can generate SCFAs (short-chain fatty acids) from endogenous mucinoglycans and dietary fibres; thus, *Muribaculaceae* may be a good candidate for “next-generation probiotics” promoting benefits through a variety of mechanisms [49].

In addition, GSF significantly decreased the abundances of *Akkermansia*, *B. caecimuris*, and the *UBA1819* genus. In many studies, the DSS treatment was characterised by an increase in *Akkermansia* [30,52]. Despite its positive role in the metabolic context [53], *Akkermansia* may play a negative role in other circumstances. Diets rich in sugar but low in fibre favour the expansion of *Akkermansia*, accelerate erosion of the intestinal mucus layer, and exacerbate colitis [54]. In another context, supplementing with *A. muciniphila* can aggravate radiation-induced intestinal injury [55]. In our study, the GSF in the DSS context alleviated DSS-related effects while reducing *Akkermansia* levels. Similar results were obtained in the DSS model with a probiotic intervention using *Lactiplantibacillus fermentum* [52]. Supplementing fibres in the GSF treatment may help reduce *Akkermansia*, thereby limiting translocation and gut inflammation [54]. *B. caecimuris* is another bacterial species significantly increased by the DSS treatment and regulated by the GSF. *B. caecimuris* was strictly correlated with injury of the distal colon caused by intestinal epithelial mitochondrial dysfunctions [56]. *B. caecimuris* displays an early, persistent rise in abundance during colonic metabolic damage [57]. The *UBA1819* genus has been increased in patients presenting various health failures, such as in patients undergoing haemodialysis, which was associated with renal complications, including anaemia [58], and in patients with diabetes and CKD, in which *UBA1819* abundance was positively correlated with the estimated glomerular filtration rate [59]. *UBA1819* is also associated with multiple sclerosis [60].

Some plant extracts, such as a traditional medicinal plant rich in flavanols, called *Epimedium*, reduced *UBA1819* in broilers, implicating an improvement in the intestinal microbiota balance, resulting in healthier microbiota [61].

The modulations of the microbiota were translated to the metabolomic level. Faecal VOCs have been found to differ in gastrointestinal diseases as well as intestinal infection [62]. Acetone is the simplest and smallest ketone produced by cells, but bacteria can also produce acetone via their metabolism. In this study, we observed a significant increase in acetone levels in faecal samples from mice in the DSS group compared with the DSS-GSF group. The presence of acetone in faeces has been correlated with specific gut taxa in IBD and cystic fibrosis [62,63]. Moreover, acetone induced the production of pro-inflammatory factors, such as IL-1β, IL-6, and COX-2, in the RAW264 murine macrophage cell line and, consequently, could trigger low-level chronic inflammation.

Elevated ketone bodies, including acetone, are implicated in oxidative stress and inflammation in various tissues [64]. This oxidative and inflammatory response to endogenous ketone bodies, including acetone, has never been described in the DSS model. Moreover, in our study, GSF increases intestinal indole production, perhaps also its derivatives, but they are often difficult to analyse. Indole can help strengthen the epithelial barrier function and reduce DSS-induced damage [65]. Recent work in 2025 shows that the indole metabolite indole-3-propionic acid reprograms T-cell mitochondrial metabolism and lowers ROS, providing an additional anti-inflammatory mechanism for microbiota-derived indoles [47]. Interesting, *Muribaculum*, increased with the GSF, breaks down dietary tryptophan into indole and its derivatives (such as indole-3 propionic acid, IPA) via tryptophanase or related enzymes encoded by its genome [49,66]. Moreover, tryptophan metabolites produced by *Muribaculum* can inhibit microglial inflammatory responses, transforming pro-inflammatory M1 macrophages into anti-inflammatory M2 macrophages [66]. In addition, [67] found that *Ruminococcus bromii*, a fibre degrader, alleviates constipation in mice and increases indole levels in the colon. In our study, we found that *Ruminococcus* increased with GSF supplementation (DSS-GSF group) and observed a positive correlation between indole production and *Ruminococcus*. Other significant correlations were observed in our study. In fact, in the colon, *Muribaculaceae* correlated negatively with IL-6/Inf-γ/F4-80 gene expression and with acetone production, and showed positive correlations with colon length and IL-10. These results, in accordance with other authors [51,68], indicated a positive effect of *Muribaculaceae* on gut homeostasis. On the contrary, *Akkermansia* and *B. caecimuris* were discovered to be negatively correlated with colon length and IL-10 and positively with acetone, IFNγ and F4/80, indicating that GSF helped to reorganise microbiota towards a positive direction, thereby inhibiting inflammation. Furthermore, our study demonstrates the usefulness of acetone, a VOC, as an indicator of inflammatory parameters and the microbiota.

This GSF-supplemented DSS model brings several innovative insights into gut–liver inflammation. It shows that a complex, fibre- and polyphenol-rich grape by-product can simultaneously modulate epithelial damage, immune gene expression, microbiota composition, and microbial metabolite profiles. Beyond the expected anti-inflammatory and antioxidant effects, the model reveals that GSF attenuates the expansion of potentially harmful taxa such as *Akkermansia* and *Bacteroides caecimuris*, reduces acetone production, and enhances indole production, thereby supporting barrier integrity and mucosal homeostasis. The coordinated protection observed in the colon, liver, and mesenteric lymph nodes underscores GSF’s capacity to act along the gut–liver–immune axis rather than only at the local colonic level.

This study opens perspectives for future work. We used only one GSF formulation at 10%, which does not allow us to disentangle the respective contributions of polyphenols, fibres, and lipids/lipophilic compounds. Additional groups receiving dephenolised GSF, or delipidated GSF, would help clarify which components are essential for the anti-inflammatory and microbiota-modulating effects. It is also very interesting to study the presence of NEPs in GSF, because some classes of polyphenols can be trapped within the fibres [69]. In future studies, we can further investigate the mechanisms by which GSF promotes indole and its derivative metabolites by dosing and characterising indole metabolites, isolating and/or identifying different *Muribaculaceae* and *Ruminococcus* strains at the species level, and validating in vitro tryptophan metabolite profiling of cultured strains.

Finally, although a 10% GSF diet is clearly effective in mice, its direct translation to humans is complicated by the dosage, palatability, and long-term adherence. For human applications, standardised, compositionally defined, and higher-concentration polyphenolic extracts, such as commercial grape seed polyphenol products (e.g., Biombalance-type formulations), are more realistic candidates. These preparations could capture much of the beneficial signalling activity observed with GSF while remaining compatible with human dietary habits and clinical trial protocols. However, this type of supplement may be useful for animals as a dietary supplement, given its potent anti-inflammatory action.

## 4. Materials and Methods

### 4.1. Grape Seed Analysis

The grape seeds used in this study are by-products of the vinification of a Tunisian Carignan (*Vitis vinifera* L.) wine, harvested during the 2021 vintage season, in the region of Grombalia, Nabeul, Tunisia. After maceration, the seeds were separated from the skin, then dried at 40 °C until the moisture content reached 5–8% and then ground into a fine flour. The polyphenol (PP) compound extraction was performed using a mixture of acetone, methanol, and water at a ratio of 7:7:6 (*v*/*v*/*v*) according to [70], Ayoub et al., 2016. Grape seed flour (1 g) was mixed with 20 mL of solvent mixture and then sonicated for 20 min in an ultrasonic bath (Branson Ultrasonics Corporation, Brookfield, CT, USA) at room temperature (RT), operating at a frequency of 40 kHz. The supernatant was collected by centrifugation (15 min at 2200× *g*), and the resulting residue was extracted twice more under the same conditions. The collected organic phases were pooled and evaporated at 35 °C under reduced pressure using a rotary evaporator (Hei-Vap Advantage, Heidolph, Schwabach, Germany). The final aqueous extract was lyophilised (Telstar, LyoQuest Azbil Telstar Technologies S.L.U., Terrassa, Spain) and stored at −20 °C until analysis.

#### 4.1.1. Characterisation and Quantification of Phenolic Compounds

An aliquot of the dried polyphenolic extract (corresponding to 30 mg) was dissolved in 3 mL of a MeOH/H_2_O/HCOOH mixture (50/50/0.1, *v*/*v*/*v*). The solutions were filtered through a 0.45 µm PTFE filter before analysis. Analyses were performed in triplicate.

UHPLC analyses were performed on an Agilent 1290 Infinity LC system (Agilent Technologies, Agilent, Santa Clara, CA, USA) with a UV/visible diode-array detector (DAD) coupled to an Esquire 6000 ion trap mass spectrometer using an ESI source (Bruker-Daltonics, Billerica, MA, USA). Chromatographic separation was performed on a Zorbax SB-C18 column (2.1 × 100 mm, 1.8 µm, Agilent).

Non-anthocyanin polyphenols were eluted using a linear gradient with a flow rate of 0.4 mL/min. The mobile phases consisted of solvent A (water/formic acid, 99.9/0.1, *v*/*v*) and solvent B (acetonitrile/formic acid, 99.9/0.1, *v*/*v*). The elution gradient for solvent B was 7% (0–0.4 min), 7–14% (0.4–3 min), 14–35% (3–8 min), 35–50% (8–8.8 min), 50–100% (8.8–9 min), 100% (9–10 min), 100–7% (10–10.2 min). Mass spectrometry analyses were carried out in the negative mode with a range of *m*/*z* 100–1450. Nitrogen was used as the drying gas at 10 L min^−1^, with a nebuliser pressure of 40 psi and a temperature of 365 °C. Capillary voltage was 3400 V, capillary exit voltage was −115.3 V, skimmer voltage was −40 V, and trap drive was 50.7. The compounds were quantified using UV detection.

Anthocyanins were eluted at a flow rate of 0.4 mL/min. The mobile phase consisted of solvent A (water/acetonitrile/formic acid, 870/30/100; *v*/*v*/*v*) and solvent B (water/acetonitrile/formic acid, 400/500/100; *v*/*v*/*v*) (Supplementary Tables S1 and S2; Figures S5 and S6). The elution gradient for solvent B was 6–30% (0–3 min), 30–50% (3–6 min), 50–60% (6–7 min), 60–65% (7–8 min), 65–100% (8–8.2 min), 100% (8.2–9.2 min), 100–6% (9.2–9.4 min). Mass spectrometry analyses were carried out in the positive mode with a range of *m*/*z* 100–1200. Nitrogen was used as the drying gas at 10 L.min^−1^ with nebuliser pressure at 40 psi and temperature at 365 °C. Capillary voltage was −3700 V, capillary exit voltage was 112.2 V, skimmer voltage was 40 V, and trap drive was 45.1. The compounds were quantified using UV detection, and results were expressed as malvidin-3-O-glucoside equivalents [30,71].

#### 4.1.2. Antioxidant Capacities In Vitro

The oxygen radical absorbance capacity (ORAC) was measured according to the manufacturer’s protocol using the BQC ORAC Assay Kit (Bioquochem S.L., Oviedo, Spain), and the assay was performed in triplicate. This kit assesses the antioxidant potential by monitoring the protection of fluorescein against oxidative degradation by peroxyl radicals generated by the free-radical initiator AAPH (2,2′-azobis-2-methylpropanimidamide dihydrochloride). The presence of antioxidants in the sample delayed the decline in fluorescein fluorescence, reflecting their capacity to neutralise free radicals. Results are expressed in micromoles of Trolox equivalents per gram of dry sample weight [30].

#### 4.1.3. Lipophilic Compounds and Fibre Content Determination

The grape seed oil was obtained by cold pressing using a screw press (FARMET DUO, FARMET, Česká Skalice, Czech Republic) at a temperature not exceeding 60 °C. The same batch of Carignan grape seeds was used to prepare the GSF analysed in this study, ensuring compositional consistency between the oil and flour fractions. The extracted oil was then stored at 4 °C under a nitrogen atmosphere to preserve its quality. Total and insoluble dietary fibres were measured using a Megazyme assay kit, in accordance with the standardised methods outlined by the AACC (32-07.01) and AOAC (991.43) protocols (Megazyme, Wicklow, Ireland). This approach ensures accurate quantification of dietary fibres in the samples without interference from other components [72].

### 4.2. Diets, Reagents and Experimental Design

Grape seeds were ground to obtain a fine flour. The standard diet (SD) was purchased from SAFE (France). The grape seed flour diet (GSFD) was prepared by supplementing the SD with 10% (*w*/*w*) GSF. DSS (2.75% *w*/*v*, colitis grade, 36,000–50,000 Da; MP Biomedicals, Illkirch-Graffenstaden, France) was used to induce experimental colitis in mice.

#### 4.2.1. Animals and Treatments

Animal experiments were carried out in accordance with the French rural and maritime fisheries codes R.214-87 to R.214-126. The Ethics Committee for Animal Experimentation at the University of Bordeaux approved the protocol (APAFIS#17304-201810191064539, 2019–2024).

Six-week-old male BALB/c mice were obtained from JANVIER LABS (Lorient, France) and acclimated for one week before the start of the experiment. Animals were kept in controlled environmental conditions (22 ± 2 °C, 60% relative humidity, 12 h light/dark cycle) with free access to food and water. Environmental enrichment (gnawing sticks and climbing structures) was provided to promote both physical activity and mental stimulation. Animals were monitored daily to assess overall health and well-being.

Mice were randomly assigned to four experimental groups (n = 6 per group):

- **Control group (CRL)** Mice received an SD (SAFE A04, SAFE, Augy, France) and normal drinking water throughout the experiment.
- **GSF group (GSF)** Mice were fed an SD supplemented with 10% GSF (*w*/*w*) for 19 days and received normal drinking water.
- **DSS group (DSS)** Mice were fed the SD and, after 10 days, colitis was induced by administering DSS (2.75% *w*/*v*) in the drinking water for 7 consecutive days, followed by 2 days with normal drinking water.
- **DSS + GSF group (DSS-GSF)** Mice were fed the SD supplemented with 10% GSF (*w*/*w*) for 10 days. Colitis was then induced, as in the DSS group, by providing 2.75% (*w*/*v*) DSS in the drinking water for 7 days, followed by 2 days with normal drinking water.

Body weight and clinical signs were recorded daily. At the end of the experiment (day 10 after DSS administration), mice were euthanised by cervical dislocation, and samples were collected for further analyses.

#### 4.2.2. Evaluation of Disease Activity Index

During the DSS treatment cycle, a disease activity index (DAI) score was used to assess disease activity in colitis. The composite DAI was calculated as the arithmetic mean of the three individual scores (body weight loss score + stool consistency score + rectal bleeding score), yielding a final value ranging from 0 (no disease) to 4 (severe disease), as previously described [30]. Body weight loss was scored as follows: 0 for no weight loss relative to the initial weight; 1 for 1–5% weight loss; 2 for 5–10% weight loss; 3 for 10–20% weight loss; and 4 for greater than 20% weight loss. Stool consistency was assessed with 0 for normal (solid pellet), 1 for a soft but pellet-shaped stool, 2 for a loose stool with some solidity, 3 for a loose stool with signs of a liquid consistency, and 4 for watery diarrhoea. Rectal bleeding was scored as 0 for no blood, 1 for no bleeding, 2 for slight bleeding, 3 for bloody diarrhoea, and 4 for gross bleeding [16,28,73].

#### 4.2.3. Histopathological Analysis

The freshly harvested distal colonic tissue was promptly fixed in 4% *v*/*v* formaldehyde for 24 h, then washed and preserved in 70% *v*/*v* ethanol at 4 °C. Tissue sections with a thickness of 3 μm from paraffin-embedded tissues (PETs) were processed for Hematoxylin/Eosin (H&E) staining Sigma, Taufkirchen, Germany, following previously described standard procedures [30]. The areas of inflammatory lesions were evaluated microscopically (Leica Microsystems GmbH, Wetzlar, Germany) and scored. The severity of inflammation was rated as follows: 0 indicated no signs of inflammation; 1 represented slight, scattered infiltrates; 2 corresponded to moderate, merging, or dispersed inflammation; and 3 signified extensive, integrating areas of inflammation. The vertical extent of inflammation was scored as 0 (none), 1 (mucosal involvement), 2 (both mucosa and submucosa), and 3 (inflammation affecting the entire wall). Hyperplasia was evaluated on a scale ranging from 0 (normal tissue) to 4 (crypt heights exceeding four times normal), with or without adenomatous polyps. Crypt damage severity ranged from 0 (no damage) to 4 (loss of crypts and the overlying epithelium). In contrast, the longitudinal extent of crypt damage was scored from 0 (no damage) to 4 (damage affecting more than 76% of the assessed area).

### 4.3. Determination of Inflammatory Markers Using qRT-PCR

Distal colon, liver, and MNL samples were harvested and immediately immersed in RNAlater (Qiagen, Hilden, Germany), incubated at 4 °C for 24 h, and then transferred to −20 °C until RNA extraction. Total RNA was extracted using the RNeasy Mini Kit (Qiagen, Germany) with an additional DNase digestion step (Thermo Scientific, Waltham, MA, USA), following the supplier’s protocol, and RNA concentration was determined with a NanoDrop One spectrophotometer (Thermo Fisher Scientific, Waltham, MA, USA). For cDNA synthesis, 250 ng of total RNA was reverse-transcribed using a hexamer-based kit (SuperScript IV, Thermo Scientific, USA). Quantitative PCR was carried out on a CFX96 system (Bio-Rad, San Francisco, CA, USA) using the SsoAdvanced™ Universal SYBR Green Supermix kit (Bio-Rad, USA). Target gene expression was normalised to the reference gene hypoxanthine–guanine phosphoribosyl transferase (HPRT). Primer sequences and cycling conditions are reported in Supplementary Table S4. Relative gene expression levels were calculated using the 2^−ΔΔCT^ method [16].

### 4.4. Microbiota Analysis

#### 4.4.1. Stool Sampling and Faecal DNA Extraction

The day before the end of the protocol, stool samples were collected in sterile, DNase-free tubes and stored at −80 °C. Around 100 mg of faeces was accurately weighed using an analytical balance (Sartorius, Göttingen, Germany) and homogenised in Tris-EDTA buffer (Tris 0.1 mM, pH 8; EDTA 1 M (Sigma, Taufkirchen, Germany)) (1 mL of buffer for 200 mg of faeces). Lysozyme (1:100, 300 mg/mL (Sigma)) was added to the mixture, and it all was incubated at 37 °C for one hour. Then, 200 µL of the mix was used in the DNA isolation kit, which was realised with the NucleoSpin^®^ Soil kit (Macherey-Nagel, Düren, Germany) according to the manufacturer’s instructions. DNA concentration and purity were determined using the NanoDrop One (Thermo Fisher Scientific, USA) [74].

#### 4.4.2. Microbiota Analysis by Illumina Sequencing

The 16S rRNA gene V3–V4 region was amplified by PCR using the forward primer 338F (5′-ACTCCTACGGGAGGCAGCA-3′) and the reverse primer 806R (5′-GGACTACHVGGGTWTCTAAT-3′) to prepare Illumina libraries. The reaction mixture, containing 10 ng of DNA, 10 μM of each primer, and PCR-grade water in a final volume of 50 μL, was prepared using the MP Taq DNA Polymerase kit (Q-Bio DNA polymerase, MP Biomedicals, Illkirch-Graffenstaden, France). PCR cycling involved a denaturation step at 95 °C for 5 min, followed by 30 cycles of 30 s at 95 °C, 30 s at 52 °C, and 45 s at 72 °C, concluding with a final extension step at 72 °C for 2 min. The quality and the quantity of the PCR product were assessed via 1% agarose gel electrophoresis. The DNA amplicons were sequenced on the Illumina MiSeq platform (Genotoul, France) as described by [22]. Read quality was assessed using GALAXY FROGS [36,75] to eliminate low-quality sequences and those with incorrect amplicon lengths. Assembled paired-end reads were clustered into operational taxonomic units (OTUs) using the Swarm algorithm with aggregation parameters of d = 1 and d = 3, following previously established protocols. A quantity of OTUs was retained for downstream analysis, after which they were classified using the SILVA 138 reference database with a quality score of 100. Samples were categorised by treatment group and normalised using DESeq2. Diversity within samples was assessed at the genus level using alpha diversity indices, including Shannon, Simpson, and inverse Simpson, calculated with SHAMAN [37]. Heatmaps, principal coordinate analysis (PCoA), and additional statistical analyses were performed using the SHAMAN platform to assess differences in abundance among samples [37].

#### 4.4.3. PICRUSt (Phylogenetic Investigation of Communities by Reconstruction of Unobserved States) Analysis

To predict the functional capacity of the gut microbiota, we analysed 16S rRNA gene expression profiles using PICRUSt2 (v2.4) based on OTU tables generated by FROGS. This computational approach models functional profiles, enabling inference of metabolic pathways, gene families, and enzyme activities within microbial communities. We emphasised MetaCyc pathway analysis at an advanced (tier 3) resolution to examine metabolic differences across experimental groups. Annotated pathways were established by linking EC numbers to entries in the MetaCyc reference database, an openly available alternative to KEGG.

### 4.5. Analysis of Volatile Organic Compounds (VOCs)

Approximately 100 mg of freshly collected faecal samples from each animal was transferred to sterile vials and immediately frozen at −20 °C until analysis. Before analysis, headspace solid-phase microextraction (HS-SPME) was performed. The SPME Fibre (75 µm CARS/PDMS, Fused Silica, Supelco, Bellefonte, PA, USA) was exposed to the headspace of each vial at 60 °C for 30 min to adsorb volatile compounds. The SPME fibre was then thermally desorbed in the gas chromatograph (GC) injector at 250 °C for 1 min [76].

Volatile metabolites were analysed with a QP 2010 SE GC/MS system (Shimadzu Corporation, Kyoto, Japan) equipped with a ZB-5MS capillary column (30 m × 0.25 mm ID, 0.25 μm, Phenomenex, Torrance, CAa, USA). Helium is used as the carrier gas at a constant flow rate of 1.0 mL/min. The oven temperature program started at 40 °C (held for 6 min), then increased to 220 °C at 5 °C/min, and was held for 5 min. The MS was operated in electron ionisation (EI) mode at 70 eV, with a source temperature of 230 °C and a scan range of *m*/*z* 35–500.

Compound identification was performed by comparing their MS spectra and retention times with those of the standard mixture, and quantification was performed using a calibration curve established with standards.

### 4.6. Statistical Analysis

Data were compared using a two-way ANOVA followed by Tukey’s multiple comparison tests. Results were presented as the mean  ±  SEM, with a *p* value of less than 0.05 indicating significance.

Tukey’s multiple-comparison post hoc test was carried out using GraphPad Prism software (version 8.2.3, GraphPad Software, LLC, Boston, MA, USA).

Alpha diversity indices and differential abundance analyses of the gut microbiota were computed and visualised using the SHAMAN platform (online version, https://shaman.pasteur.fr/, accessed on 2 February 2026, Institut Pasteur, Paris, France).

For PICRUSt, abundances were standardised to z-scores and then analysed with ANOVA, followed by a Tukey post hoc test, using the SHAMAN platform (online version, https://shaman.pasteur.fr/, Institut Pasteur, Paris, France) [37].

## 5. Conclusion

The present study demonstrates that diet supplementation with GSF, a fibre and polyphenol-rich winemaking by-product, exerts multimodal protection against DSS-induced acute colitis in mice. Our findings document significant attenuation of colonic epithelial injury, and favourable modulation of immunoinflammatory markers along the intestine–liver–mesenteric lymph node axis. Beneficial reshaping of gut microbiota composition, including enrichment of butyrate-producing taxa such as *Muribaculaceae* and *Ruminococcus*, and partial normalisation of faecal microbial metabolite profiles, notably reduced acetone and elevated indole levels, the latter being known to support epithelial barrier integrity.

From a translational standpoint, these preclinical results provide a rational scientific basis for designing pilot studies in humans. GSF can be a strong antioxidant and a source of positive fibres, at low cost, with valorisation of the wine-making west. However, it is essential to emphasise that the dose used in the present study (10% GSF in the diet) cannot be directly extrapolated to humans without rigorous dose adaptation.

This work also opens new research avenues for the identification of the specific bioactive fractions of GSF (polyphenols, fibres, PUFAs) and for the mechanistic characterisation of bacterially derived tryptophan metabolites—in particular, IPA—in relation to mucosal immune regulation along the gut–liver axis.

## Figures and Tables

**Table 1:** Main GSF identified phenolic compounds.

Retention Time (min)	Molecular Mass (Da)	[M − H]^−^	Fragment Ions in Negative Mode	Proposed Structure	Content (µg/g) GSF Extract
1.2	170	169	125	Gallic acid ^a^	1766 ± 64
2.2	154	153	109	Protocatechuic acid ^b^	595 ± 23
3.0	578	577	451, 425, 407, 289	Type B procyanidin dimer ^d^	327 ± 6
3.5	290	289	245, 205	Catechin ^e^	2234 ± 77
3.9	230	229	185, 141, 115	Unidentified compound	Not quantified
4.1	578	577	451, 425, 407, 289	Procyanidin B2 ^d^	690 ± 34
4.3	230	229	185, 141, 115	Unidentified compound	Not quantified
4.5	290	289	245, 205	Epicatechin ^f^	1152 ± 38
4.9	866	865	739, 695, 577, 407, 289	Type B procyanidin trimer ^d^	378 ± 16
5.1	730	729	711, 603, 559, 451, 441, 407, 289	Type B-gallate procyanidin dimer ^d^	611 ± 14
5.4	164	163	119	Coumaric acid ^g^	98 ± 4
5.9	464	463	301, 179, 151	Quercetin-O-hexoside ^h^	205 ± 3
5.9	442	441	289	Epicatechin gallate ^f^	772 ± 38
8.4	302	301	179, 151	Quercetin ^h^	125 ± 6

(a) quantified as gallic acid equivalents at 280 nm; (b) quantified as protocatechuic acid equivalents at 260 nm; (c) quantified as tryptophan equivalents at 280 nm; (d) quantified as procyanidin B2 equivalents at 280 nm; (e) quantified as catechin equivalents at 280 nm; (f) quantified as epicatechin equivalents at 280 nm; (g) quantified as p-coumaric acid equivalents at 310 nm; (h) quantified as quercetin equivalents at 360 nm.

**Table 2:** Major GSF-identified anthocyanins.

Retention Time (min)	Molecular Mass (Da)	M^+^	Fragmentation in Positive Mode	Proposed Structure	Content (µg/g) GSF Extract
3.5	493	493	331	Malvidin-3-O-glucoside ^a^	114.78 ± 6.08
4.9	611	611	303	Delphinidin-3-O-(6-O-p-coumaroyl)-glucoside ^a^	48.06 ± 2.40
5.3	535	535	331	Malvidin-3-O-(6-O-acetyl)-glucoside ^a^	80.04 ± 3.98
5.7	625	625	317	Petunidin-3-O-(6-O-p-coumaroyl)-glucoside ^a^	76.34 ± 3.97
6.3	609	609	301	Peonidin-3-O-(6-O-p-coumaroyl)-glucoside ^a^	132.06 ± 3.30
6.5	639	639	331	Malvidin-3-O-(6-O-p-coumaroyl)-glucoside ^a^	849.99 ± 22.82

(a) quantified as malvidin-3-O-glucoside equivalents at 520 nm. Among the identified compounds, Malvidin-3-O-(6-p-coumaroylglucoside) is the major anthocyanin, reaching the highest concentration (0.845 mg/g of extract). It is an acylated malvidin derivative, in which the glucose moiety is esterified by p-coumaric acid at position 6. This structure increases pigment stability and enhances colour intensity, especially at a pH similar to that of wine. Other malvidin derivatives, as well as delphinidin-, petunidin-, and peonidin-3-O-(6-p-coumaroylglucoside), are present in smaller quantities but help broaden the anthocyanin spectrum. This profile agrees with reports describing malvidin-3-O-(6-p-coumaroylglucoside) as a key pigment in red wine grapes and their by-products [34].

**Table 3:** Chemical composition of GSF.

Parameter	Value	Unit
Lipophilic compounds (fats) in GSF	9.5	% (*w*/*w*)
Fibre content in GSF	64.0	% (*w*/*w*)

## Data Availability

The original contributions presented in this study are included in the article/Supplementary Material. Further inquiries can be directed to the corresponding author.

## Supplementary Materials

The following supporting information can be downloaded at https://www.mdpi.com/article/10.3390/ph19081189/s1. Table S1: Gradient program used for separating phenolic compounds (excluding anthocyanins) by UPLC DAD MS; Table S2: Gradient program used for the separation of anthocyanins by UPLC-DAD-MS; Table S3: Correlation tables; Table S4: RT-qPCR primer sequences; Figure S1: Log2 fold changes in microbial phylum level; Figure S2: Log2 fold changes in microbial family level; Figure S3: Log2 fold changes in microbial species level; Figure S4: 16S rRNA gene V3–V4 Gel; Figure S5:UPLC-DAD chromatogram at 280 nm; Figure S6: UPLC-DAD chromatogram at 520 nm.

## Abbreviations

**IL-6**	Interleukin-6
**TNF-α**	Tumor necrosis factor alpha
**IL-23**	Interleukin-23
**IL-10**	Interleukin-10
**CXCL1**	C-X-C motif chemokine ligand 1
**TGF-β**	Transforming growth factor beta
**FOXP3**	Forkhead box P3
**F4/80 (Adgre1)**	F4/80 antigen (adhesion G protein-coupled receptor E1)
**iNOS**	Inducible nitric oxide synthase
**CAT**	Catalase
**SOD1**	Superoxide dismutase 1
**ZO-1**	Zonula occludens-1
**OCLN**	Occludin
