# Supplementary material for: Prevention of Intestinal Inflammation and Gut Dysbiosis by Prebiotic Grape Seed Flour in Mice with DSS-Induced Colitis
Source: Pharmaceuticals (Basel). 2026 Jul 29;19(8):1189. doi: 10.3390/ph19081189 (PMC13516961; doi:10.3390/ph19081189)
Supplement: Supplementary file 1 [file pharmaceuticals-19-01189-s001.zip › Figure S4.pdf]

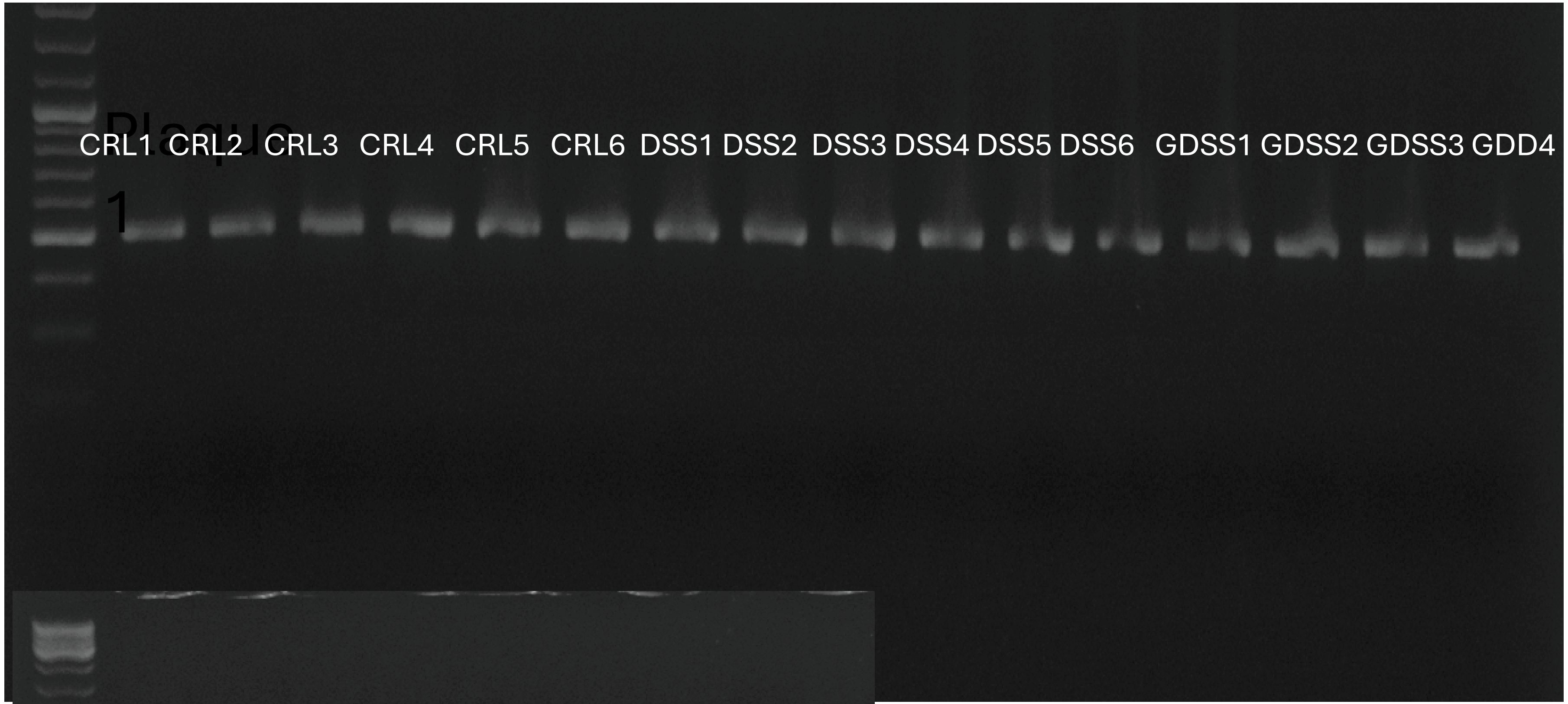
Plaque 1
CRL1 CRL2 CRL3 CRL4 CRL5 CRL6 DSS1 DSS2 DSS3 DSS4 DSS5 DSS6 GDSS1 GDSS2 GDSS3 GDD4

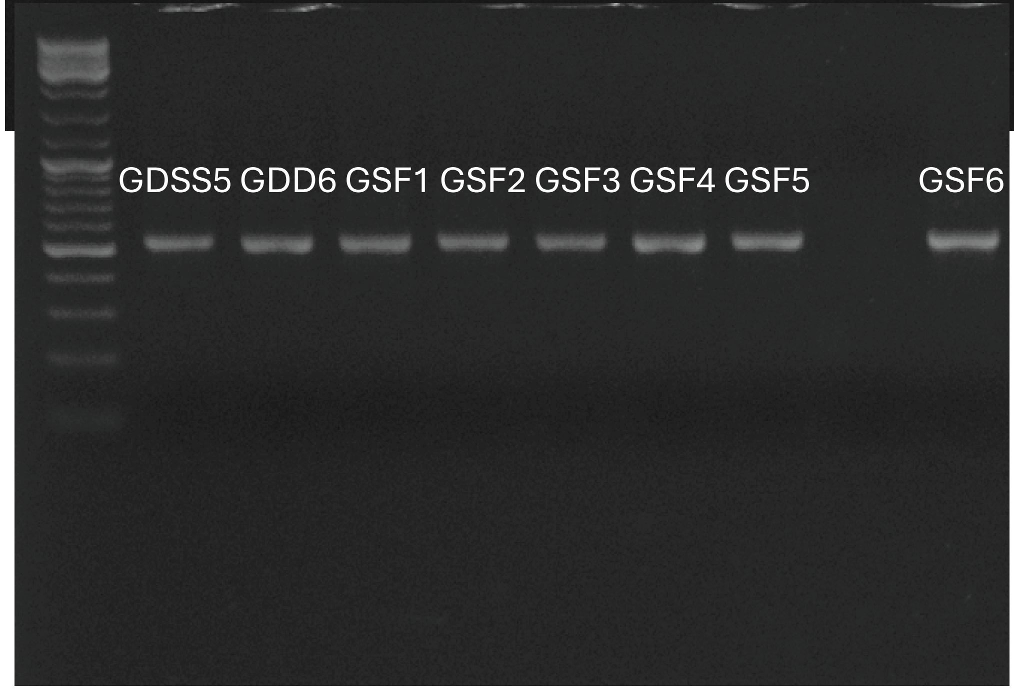
GDSS5 GDD6 GSF1 GSF2 GSF3 GSF4 GSF5 GSF6
