# Supplementary material for: Prevention of Intestinal Inflammation and Gut Dysbiosis by Prebiotic Grape Seed Flour in Mice with DSS-Induced Colitis
Source: Pharmaceuticals (Basel). 2026 Jul 29;19(8):1189. doi: 10.3390/ph19081189 (PMC13516961; doi:10.3390/ph19081189)
Supplement: Supplementary file 1 [file pharmaceuticals-19-01189-s001.zip › Figure S5.pdf]

At the outlet of the diode-array detector, the eluent was introduced into the mass spectrometer. Analyses were performed in negative and/or positive ion mode. LC-MS spectra were acquired in full-scan mode over an m/z range from 100 to 1400. All data were then collected and processed using Hystar software, version 3.0.

C). Characterization and quantification of major phenolic compounds (excluding anthocyanins) by UPLC-DAD-MS

The UPLC-DAD chromatogram of the extract recorded at 280 nm is shown in Figure 1 below.

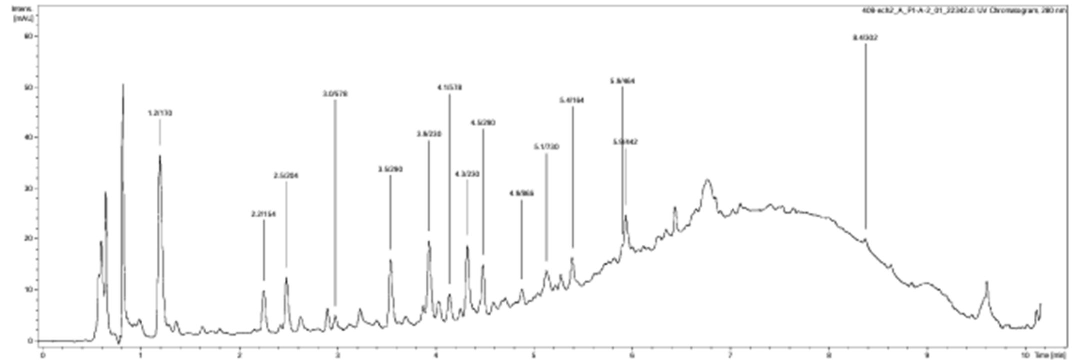

Figure S5. UPLC-DAD chromatogram at 280 nm.
