# Supplementary material for: Prevention of Intestinal Inflammation and Gut Dysbiosis by Prebiotic Grape Seed Flour in Mice with DSS-Induced Colitis
Source: Pharmaceuticals (Basel). 2026 Jul 29;19(8):1189. doi: 10.3390/ph19081189 (PMC13516961; doi:10.3390/ph19081189)
Supplement: Supplementary file 1 [file pharmaceuticals-19-01189-s001.zip › Figure S6.pdf]

C) Characterisation and quantification of major anthocyanins by UPLC-DAD-MS

The UPLC-DAD chromatogram of the extract recorded at 520 nm is shown in Figure 2 below.

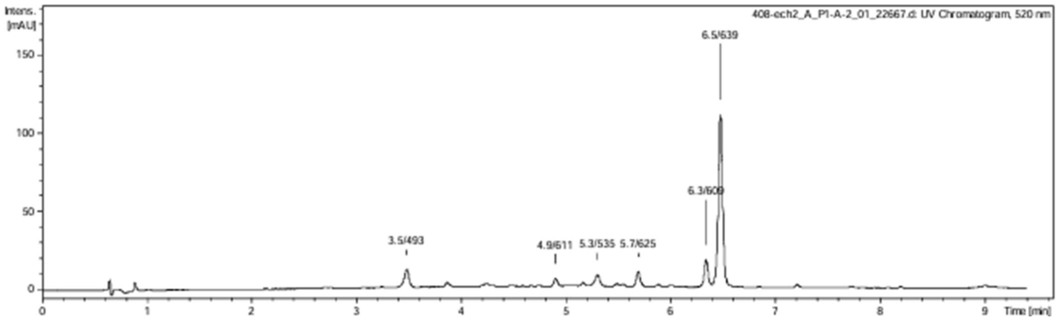

Figure S6. UPLC-DAD chromatogram at 520 nm.
