# Supplementary material for: Prevention of Intestinal Inflammation and Gut Dysbiosis by Prebiotic Grape Seed Flour in Mice with DSS-Induced Colitis
Source: Pharmaceuticals (Basel). 2026 Jul 29;19(8):1189. doi: 10.3390/ph19081189 (PMC13516961; doi:10.3390/ph19081189)
Supplement: Supplementary file 1 [file pharmaceuticals-19-01189-s001.zip › Table S1.pdf]

A)The mobile phases used were: A, water containing 0.1% formic acid; and B, acetonitrile containing 0.1% formic acid. The flow rate was set at 0.4 mL/min. The following gradient program was used to separate phenolic compounds.

Time (min)	% Solvent B
0	7
0.4	7
3	14
8	35
8.8	50
9	100
10	100
10.2	7

Table S1. Gradient program used for separating phenolic compounds (excluding anthocyanins) by UPLC-DAD-MS.
