# Supplementary material for: Prevention of Intestinal Inflammation and Gut Dysbiosis by Prebiotic Grape Seed Flour in Mice with DSS-Induced Colitis
Source: Pharmaceuticals (Basel). 2026 Jul 29;19(8):1189. doi: 10.3390/ph19081189 (PMC13516961; doi:10.3390/ph19081189)
Supplement: Supplementary file 1 [file pharmaceuticals-19-01189-s001.zip › Table S2.pdf]

B) Characterisation and quantification of anthocyaninsThe mobile phases used were: A, water/acetonitrile/formic acid (870/30/100, v/v/v) and B, water/acetonitrile/formic acid (400/500/100, v/v/v). The flow rate was 0.4 mL/min. The following gradient program was used to separate anthocyanins.

Time (min)	% Solvent B
0	6
3	30
6	50
7	60
8	65
8.2	100
9.2	100
9.4	6
12.4	6

Table S2. Gradient program used for the separation of anthocyanins by UPLC-DAD-MS.
