# Supplementary material for: Prevention of Intestinal Inflammation and Gut Dysbiosis by Prebiotic Grape Seed Flour in Mice with DSS-Induced Colitis
Source: Pharmaceuticals (Basel). 2026 Jul 29;19(8):1189. doi: 10.3390/ph19081189 (PMC13516961; doi:10.3390/ph19081189)
Supplement: Supplementary file 1 [file pharmaceuticals-19-01189-s001.zip › Table S3.pdf]

	MURIBACULACEAE	AKKERMANSIA	UBA1819	Bacteroides Ceacimuris	Ruminococcus	COLON length	IL6
COLON length	*	*** -	ns	** -			
IL6	* -	ns	ns	ns			
IL10	*	***-	ns	*			
Inf g	*-	***	ns	*			
F 4 80	**-	***	ns	**			
Acetone	*-	***	ns	***		***	***
Indole	ns	ns	ns	ns	**		

TableS3 :Table of correlations
