# Supplementary material for: Prevention of Intestinal Inflammation and Gut Dysbiosis by Prebiotic Grape Seed Flour in Mice with DSS-Induced Colitis
Source: Pharmaceuticals (Basel). 2026 Jul 29;19(8):1189. doi: 10.3390/ph19081189 (PMC13516961; doi:10.3390/ph19081189)
Supplement: Supplementary file 1 [file pharmaceuticals-19-01189-s001.zip › Table S4.pdf]

**Table S4.** RT-qPCR primer sequences.

| Gene | Sequence 5′-3′ | °C ealing T | Accesion Number |
|---|---|---|---|
| *Il-6* | FW: TACCACTTCACAAGTCGGAGGC<br>RV: CTGCAAGTGCATCATCGTTGTTC | 60 | NM_031168 |
| *Tnf-α* | FW: GGTGCCTATGTCTCAGCCTCTT<br>RV: GCCATAGAACTGATGAGAGGGAG | 60 | NM_013693 |
| *Il-23* | FW: AGCAACTTCACACCTCCCTAC<br>RV: ACTGCTGACTAGAACTCAGGC | 60 | NM_031252 |
| *Il-10* | FW: AGAAGCATGGCCCAGAAATCA<br>RV: GGCCTTGTAGACACCTTGGT | 60 | NM_010548 |
| *Cxcl1* | FW: GGGTGTTGTGCGAAAAGAAGTGC<br>RV: GGGAAGCGTCAACACGTGCG | 60 | NM_008176 |
| *Tgf-* | FW: CCGCAACAACGCCATCTATG<br>RV: CTCTGCACGGGACAGCAAT | 60 | NM_011577 |
| *Foxp3* | FW: GGTACACCCAGGAAAGACAGC<br>RV: AAGACCTTCTCACAACCAGGC | 60 | NM_054039 |
| *F4/80 (Adgre1)* | FW: CGTGTTGTTGGTGGCACTGTGA<br>RV: CCACATCAGTGTTCCAGGAGAC | 60 | NM_010130 |
| *iNOS* | FW: GAGACAGGGAAGTCTGAAGCAC<br>RV: CCAGCAGTAGTTGCTCCTCTTC | 60 | NM_010927 |
| *Cat* | FW: CGGCACATGAATGGCTATGGATC<br>RV: AAGCCTTCCTGCCTCTCCAACA | 60 | NM_009804 |
| *Sod1* | FW: AACCAGTTGTGTTGTCAGGAC<br>RV: CCACCATGTTTCTTAGAGTGAGG | 60 | NM_011434 |
| *ZO-1* | FW: GTTGGTACGGTGCCCTGAAAGA<br>RV: GCTGACAGGTAGGACAGACGAT | 60 | NM_009386.1 |
| *Ocln* | FW: TGGCAAGCGATCATACCCAGAG<br>RV: CTGCCTGAAGTCATCCACACTC | 60 | NM_008756 |

| | | | |
|---|---|---|---|
| *Muc2* | FW: ACCGCCTCACTCTGCCCCAA<br>RV: TGCAGCCACTGCCCGTGATG | 60 | NM_023566 |
| *ChREBP* | FW: CAGATGCGGGACATGTTTGA<br>RV: AATAAAGGTCGGATGAGGATGCT | 60 | NM_021455 |
| *Hprt* | FW: GTTAAGCAGTACAGCCCCAAA<br>RV: AGGGCATATCCAACAACAAACTT | 60 | NM_013556 |

Thermal Cycling Program

1. **Initial denaturation and polymerase activation**: 95°C for 5 minutes

2. **Amplification** (40 cycles):

    - Denaturation: 95°C for 10 seconds
    - Annealing: 60°C for 30 seconds
    - Extension: 72°C for 30 seconds (with plate

read) 3. **Final extension**: 72°C for 5 minutes

4. **Melt curve analysis**:

    - 65°C to 95°C with 0.5°C increments
    - 5 seconds per step
